# Biomimetic Nanomembranes: An Overview

**DOI:** 10.3390/biomimetics5020024

**Published:** 2020-05-29

**Authors:** Zoran Jakšić, Olga Jakšić

**Affiliations:** Center of Microelectronic Technologies, Institute of Chemistry, Technology and Metallurgy, University of Belgrade, 11000 Belgrade, Serbia; olgica@nanosys.ihtm.bg.ac.rs

**Keywords:** nanomembrane, bionics, nanoscience, nanotechnologies, ion channels, biosensing, biointerfaces, neural interfaces, nanofluidics

## Abstract

Nanomembranes are the principal building block of basically all living organisms, and without them life as we know it would not be possible. Yet in spite of their ubiquity, for a long time their artificial counterparts have mostly been overlooked in mainstream microsystem and nanosystem technologies, being a niche topic at best, instead of holding their rightful position as one of the basic structures in such systems. Synthetic biomimetic nanomembranes are essential in a vast number of seemingly disparate fields, including separation science and technology, sensing technology, environmental protection, renewable energy, process industry, life sciences and biomedicine. In this study, we review the possibilities for the synthesis of inorganic, organic and hybrid nanomembranes mimicking and in some way surpassing living structures, consider their main properties of interest, give a short overview of possible pathways for their enhancement through multifunctionalization, and summarize some of their numerous applications reported to date, with a focus on recent findings. It is our aim to stress the role of functionalized synthetic biomimetic nanomembranes within the context of modern nanoscience and nanotechnologies. We hope to highlight the importance of the topic, as well as to stress its great applicability potentials in many facets of human life.

## 1. Introduction

Biological nanomembranes are ubiquitous and fundamental, and no life as we know it would be possible without them. They ensure the structural integrity of cells and, in eukaryotic cells, of their organelles (including the most complex one, the nucleus) by defining their boundaries and shapes and by shielding their interior, while at the same time providing their defouling. Simultaneously with these protective functions, they enable extremely complex and selective exchange of matter between the cell/organelle and its environment. This includes highly sophisticated and balanced import and export of different matter, from ions to highly complex organic molecules, cell signaling based on receptors and many other functions. While at first glance it could seem that nanomembranes represent a simple envelope around the cell or organelle contents, in reality they are miraculously sophisticated nanomachines ensuring the very existence of life. It is not by accident that biologically functionalized nanomembranes are the most widespread and ubiquitous building block of life.

As far as the composition of biological nanomembranes is concerned, it depends on the type of the cell (prokaryotic or eukaryotic, plant or animal). The basic structure of a vast majority of cell membranes consists of phospholipid layers which are amphipathic, i.e., they simultaneously consist of a hydrophilic (or polar) head and a hydrophobic tail. When a cell membrane is formed, the hydrophobic tails are clustered together, while the hydrophilic head groups face outside of the membrane, thus forming a lipid bilayer [1,2,3], a two-molecule sheet structure with a thickness of about 4 nm. Biological nanomembranes contradict the widespread misconception that biochemical processes must occur in water-containing environments only, since very complex biochemical and biological processes take place within the soft and movable yet definitely non-aqueous structure of the cell membrane [4]. Biological nanomembranes contain various functionalizing proteins that ensure exchange of matter with the external world and which can either associate with the polar “heads” of bilayer—the so-called peripheral proteins—or with the membrane matrix—integral proteins. There is a third group of membrane proteins, the non-permanent “ambiquitous proteins”. The in-plane dynamics of the membrane system is described by the classic “fluid mosaic” model of Singer and Nicolson from 1972, which has been updated several times since, for instance by Engelmann in 1977 [4,5].

Biological cell membranes are often encased within cell walls that ensure mechanical robustness to the cell and a new level of protection, both from outside agents and inside phenomena like excessive swelling, while at the same time enabling traffic of matter to the cell and from it. They are often composed of polysaccharides. Animal cells do not have a cell wall. In the case of land plants, the wall most often consists of polysaccharide cellulose or pectin, and may incorporate lignin or cutin and some other constituents. In fungi, cell walls are often built of chitin, the same material that is encountered in the exoskeletons of arthropods. Algae also have cell walls made of polysaccharides (e.g., carrageenan and agar). In prokaryotic bacteria, cell walls are built of peptidoglycan. In Archea, the most often encountered constituents are surface layer proteins (S-proteins), glycoproteins, pseudopeptidoglycan and polysaccharides. Some single-cell algae even produce their cell walls from silicon dioxide.

It stands to reason that among the basic goals of biomimetics, especially within the framework of nanoscience, nanotechnologies and micro/nanosystems, one of the important places should belong to the synthesis and use of artificial nanomembranes, and especially those with imparted biomimetic functionalities. We have much to learn and copy from one of the most ubiquitous biological structures and one of the most perfect nanomachines in existence. Whenever possible, this is done using an extended toolbox of materials that includes both the substances that make up biological structures, and those rarely or never encountered in natural nanomembranes. The benefit of such an approach is that it helps us attain biomimetic properties that enhance or completely exceed the natural ones.

A maybe even more important goal is to ensure an artificial pathway to re-creating, enhancing and improving the already extremely rich functionalities of the biological membranes, imparted mostly by the permanently or temporarily associated proteins, including but not limited to the controlled and selective trans-membrane exchange of matter. The possibility of manipulating such fundamental properties of one of the most important building blocks of life in order to make synthetic replicas, enhanced versions, or even versions with completely novel functionalities, is of supreme importance, and the potential benefits for the quality of human life are staggering.

Multifunctionalized biomimetic nanomembranes can be used in a vast number of different fields. For instance, the property of selective transmembrane transport has already shown its value in separation science and technology [6]. Some particular application examples include wastewater treatment [7], desalination [8,9], removal of toxic chemicals [10], of bacterial and viral agents [11] and gathering and concentration of targeted species (e.g., precious metals) from seawater [12]. Another wide field of use is biomedicine and life sciences [13], and covers such diverse areas as biointerfaces including brain–machine interfaces [14], scaffolds for tissue growth [15,16], biosensors [17,18], drug delivery and targeting [19], various labs-on-a-chip [20], DNA sequencers [21], etc. A field where the benefits of biomimetic nanomembranes are already starting to lead to large advancements is renewable energy [22]. For instance, artificial ion channels have proven themselves useful for proton-exchange fuel cells [23]. In addition, there are fields where the biological functionalities are combined in completely novel ways, like in highly sensitive and selective biosensors [24,25], various passive and active optoelectronic devices [26], plasmonic structures and devices [27,28], metasurfaces [29,30], etc.

Some of the research on freestanding synthetic nanomembranes reaches as far back as to the 1930s [31], but the real advent of related investigations occurred with the discovery of graphene [32,33] and fabrication of other quasi-2D materials like silicene [34], molybdenum disulfide [35], borophene [36], phosphorene (black phosphorus), germanene [37], stanene [38], plumbene [39], MXenes [40,41,42,43,44] and other 2D materials synthesized to date [45].

The first freestanding artificial nanomembranes had only modest aspect ratios, but since 2004 the experimental designs have started to evolve rapidly and the range of available materials has been expanding ever since. Historically, the development of freestanding nanomembranes, including their fabrication, functionalization and, in recent times, application, proceeded in the direction of larger and more durable membranes, in spreading the toolbox of the available materials and the number of types of the freestanding structures and in introducing them to various field of human life. Today there is a vast number of different types of synthetic nanomembranes and mechanisms of their biomimetic functionalization. This development is continually expanding to encompass more and more fields, and the focus is gradually shifting to targeted structures that are fit for specific applications. The aim of this paper is to review this exciting and ever-expanding field, concentrating on the more recent achievements. Some topics and even whole fields are surely skipped, as well as some important teams who made immeasurable contributions to the subject of biomimetic nanomembranes. The authors are only left to say in their defense that such things unavoidably happen with basically all reviews of this kind. Of course, we did our best to avoid such occurrences. We apologize to all of those who significantly contributed to this vast field and whose work was nevertheless omitted or presented in insufficient detail or at an inadequate level.

In this review paper, we first systematically outline various types of the synthetic nanomembranes described so far, then we present a short overview of the methods for their fabrication. This is followed by a review of different approaches to nanomembrane biomimetic functionalization, together with a consideration of the main synthetic functionalizing structures drawn from biology, including, among others, artificial ion channels, ion pumps and nuclear pore complexes. The rest of the paper describes in some detail different applications of multifunctionalized biomimetic synthetic nanomembranes. We continue with a description of some possible future research directions and finish with a conclusion.

In this article, we directed our maximum efforts to render the text as exhaustive as possible and to cover as many diverse fields connected with biomimetic nanomembranes as we were able. One of the highlights is the inclusion of the most recent developments in the field. About two thirds of the references we cited were published within the last five years, while a number of them were published in 2020. Another contribution is that many topics appearing throughout this review, to the best of the authors’ knowledge, have never before been reviewed in dedicated reviews. Among the key points is the comprehensive review of synthetic structures for biomimetic functionalization.

## 2. Principles

### 2.1. Definitions and Terminology

Here we define the synthetic nanomembrane as an organic, inorganic or hybrid quasi-2D artificial freestanding or free-floating structure with a thickness below 100 nm, with the bottom limit being a monatomic/monomolecular thickness, and simultaneously having a large thickness-to-lateral size aspect ratio. “Large” here means at least 2 to 3 orders of magnitude, but much higher aspect ratios are readily achieved. If the obtained aspect ratio is one million or higher, such nanomembranes are denoted as “giant” [46]. One may notice that this definition excludes nanocomposite membranous structures with a thickness exceeding 100 nm, although there are some instances in the literature where the term “nanomembranes” is reserved for the above described structures with a thickness of up to 300 nm–400 nm. Additionally, the designation “nanomembranes” is sometimes used for much thicker structures with high aspect ratios, even those in excess of hundreds of micrometers, if they are nanostructured. A typical example would be nanoporous membranes for separation, which are also sometimes denoted in the literature as nanomembranes. 

Biomimetic nanomembranes can be defined as a subclass of synthetic nanomembranes with some of their functionalities partially or fully mimicking those of natural biological membranes. This means that we can use any material or structure that in itself is not biomimetic for their production and functionalization, for instance metals or other inorganic materials and structures such as carbon nanotubes or metalorganic frameworks, as long as they perform a function within the nanomembrane that mimics a biological one. It is not the composition, but the function that makes something biomimetic. Our definition excludes the use of natural biological structures as parts or building blocks—the biomimetic membranes and their functional parts we consider here are purely synthetic.

Freestanding nanomembranes have been variably called “interfaces without bulk” [47], “quasi-2D objects”, “freestanding films”, “self-supported films”, “unbacked films”, “suspended films”, and, depending on the type of a nanomembrane, “free-floating films”. The word “films” in all of these expressions may be replaced with “nanofilms”, “ultrathin structures”, “2D objects”, “2D structures”, or simply “membranes”.

On the other hand, the term “nanomembranes” has been used in the literature to refer to quasi-2D structures backed with some solid support or substrate. For instance, in separation science a porous nanomembrane to be used as a nanofilter or a nanosieve will often be backed with a macroporous substrate whose function is to ensure a robust support without hindering fluid flow. Despite being denoted as “nanomembranes”, such structures may have a thickness of the order of hundreds of micrometers, even a few millimeters. The term has been even sometimes been applied to fully macroscopic membranes, simply because they are nanostructured (for instance, they may have nanometer-sized pores).

### 2.2. Properties of Non-Functionalized Synthetic Nanomembranes

A biomimetic nanomembrane may be self-supported in air/vacuum or it may be positioned between two different fluids (including gas/liquid, a very frequently encountered combination). As a rule, we do not consider nanomembranes transferred and fixed to any kind of solid support.

We chose the upper thickness limit of 100 nm because simple scaling laws valid from the macroscopic world to the microsystems cease to be applicable at approximately that point. Physics and chemistry of nanomembranes often simply do not obey the scaling laws. Quantum effects become necessary for the description of charge carrier transport, heat transfer and even optical effects (the appearance of the evanescent fields and localization effects) [48]. Additionally, nanofluidics is governed by a different set of rules in comparison to either conventional fluid mechanics or even microfluidics [49]. This “nanofluidic book of rules” must be applied when describing fluid transport through nanometric pores in nanomembranes. Even mechanical properties do not remain the same—for instance, Young’s modulus is modified and becomes thickness-dependent [50]. Many surprising and counter-intuitive phenomena arise, of which we list some in the remainder of this subsection.

Counterintuitively, synthetic nanomembranes may be very robust and tough, especially bearing in mind their minuscule thickness. It has been recorded that even monatomic/monomolecular membranes are able to withstand single-sided pressures in excess of a few bar [47]. It has also been noted that 35 nm thick composite giant nanomembranes made of polymer host containing zirconium oxide are sufficiently strong to hold quantities of liquid 70,000 times heavier than the nanomembranes themselves [51].

**Figure 1:**
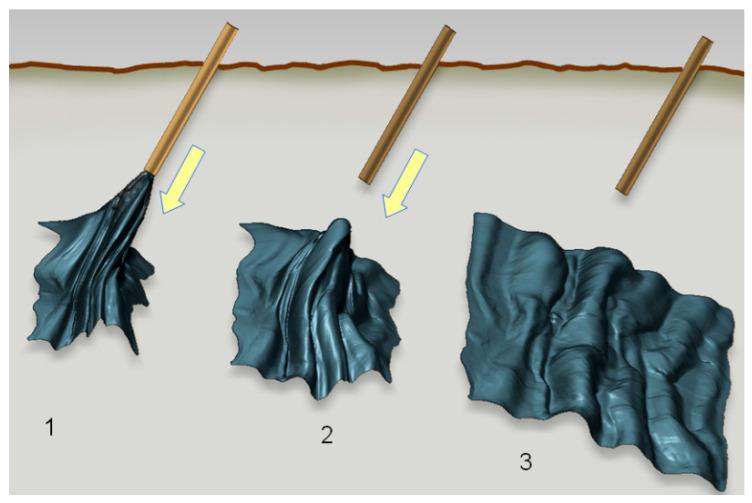
Successive steps after local administration of metal-composite nanomembrane by syringe; 1. membrane being ejected into liquid or tissue, but still partly within the needle; 2. membrane completely ejected into the fluid and beginning to unfurl; 3. fully unfurled membrane. The authors’ own work from [52].

The property of toughness is at the same time combined with extreme foldability, i.e., the flexural rigidity of freestanding nanomembranes is extremely low. This is valid even for those membranes consisting of relatively brittle materials that are easily damaged or broken if their thickness is macroscopic. For instance, Vandamme et al. [51] showed that a nanomembrane can reversibly, repeatedly and without any damage pass through a syringe with an internal diameter 30,000 times smaller than the membrane area and unfurl itself after being ejected into fluid, completely restoring its previous shape (Figure 1).

From the point of the mechanics and the theory of elasticity, external pressure is most often balanced in nanomembranes exclusively by in-plane membrane stresses, i.e., without a bending rigidity component. This stems from the theory of plates and shells [53,54], to which thick plates, diaphragms, membranes and nanomembranes all belong and conform. However, there are exceptions, which form the basis of stress engineering in nanomembranes.

Nanomembranes exhibit extremely large surface-to-volume ratios (“interfaces without volume”) [47]. This proves useful for those chemical or biomedical applications that are proportional to the active surface (e.g., catalytic or enzymatic function, drugs delivery) or require soft and pliable materials (e.g., implantable structures). A consequence of their enormous aspect ratio is that nanomembranes represent one of the rare nano-objects that can be seen by the naked eye and manually handled with only a modest degree of precautions [55].

The nanomembrane stiffness decreases with their thickness and the critical bending radius also decreases. As an example, we quote the case of silicon nanomembranes [56]. It has been shown that a brittle silicon wafer becomes foldable and stretchable at low values of thickness. A silicon membrane 300 nm thick has a critical bending radius of over 5 μm. At the same time, a silicon nanomembrane 10 nm thick has a bending radius of 500 nm [57]. Thus, biomimetic nanomembranes are highly stretchable and easily assume the shape of the surface to which they are transferred (mechanical conformability). Such low flexural rigidity makes them convenient for stretchable and foldable electronics [56].

Nanomembranes generally, not only the biomimetic ones, exhibit self-healing (self-repair) properties [58,59]. This means that small damage is spontaneously restructured and repaired. A possible explanation for this effect is that in such a low-dimensional structure, the in-plane dynamics of its constituents are very different and much higher than that in bulk.

Biomimetic nanomembranes offer the possibility of integrating bio-inspired functionalities with those seldom or never found in nature. Examples include plasmonic biomimetic nanomembranes and nanomembranous metasurfaces [27,60].

### 2.3. Types of Synthetic Nanomembranes

Synthetic membranes can be classified based on the types of material used for their fabrication. We propose one such classification in Table 1. In the further text, we proceed by elaborating further details on some of the main classes of artificial freestanding nanomembranes.

#### 2.3.1. Inorganic Nanomembranes

##### Metal Nanomembranes

The first freestanding pure metal ultrathin membranes were produced as early as 1931 (unbacked gold) [31]. Their thickness was about 200 nm. The lateral dimensions of their filament structures, however, were quite small, about 25 μm × 3 mm. The production technologies for pure metal freestanding nanomembranes evolved over years and Jia et al. reported in 2019 a routine production of large-area (up to 75 cm^2^) freestanding gold nanomembranes with arrays of nanoholes and with thickness values as low as 50 nm [61].

##### Metal Nanocomposite (Mixed Matrix) and Alloy Nanomembranes

These include constituents mixed at the atomic/molecular level or consisting of intermixed nanocrystallites. An example would be nanomembranes in which metallic alloys are their basic material [62]. Among other examples are nanomembranes that represent nanocomposites of metals with non-metallic components; e.g., in [55] chromium-based nanomembranes with giant aspect ratios (>1,000,000) containing silicon and oxygen atoms were reported.

##### Diamond

Freestanding sub-micrometer diamond membranes were reported in [63]; however, their smallest achieved thickness was 210 nm. To produce these diamond membranes, the authors used a double-implant process, followed by annealing, and finally wet etching. For many years, the reported thickness was a de facto standard for ultrathin diamond membranes [64,65,66]. However, Yoshikawa et al. succeeded in producing nanocrystalline diamond freestanding nanomembranes with the thickness values between 10 nm and 50 nm [67]. Many freestanding diamond membranes were used to fabricate 3D self-assembled forms using the methods of rolled-up nanotechnology [65]. The obtained forms included, among many others, tubes, nested tubes, rings, nested rings, nanoribbons, jagged nanoribbons, and various helical structures. Diamond nanomembranes were used for helical biomimetic structures with controllable properties in [66].

##### Diamondoids

Diamondoids [68] are diamond crystal molecules formed in the shape of cage, fused to each other, terminated by a hydrogen atom and superimposed upon diamond (bulk or thin film). The simplest diamondoid is adamantane, consisting of only one diamond cage; a higher diamondoid is tetramantane, which contains of four cages, etc. Self-assembled monolayers of tetramantane with large areas were reported in [69]. The stability of diamondoids can be enhanced by graphene.

##### Diamond-Like Carbon

Diamond-like carbon (DLC) or hard carbon is an amorphous allotrope of carbon, containing mostly sp^3^-hybridized carbon atoms. It is characterized by high hardness akin to that of diamond. Out of seven different existing forms of DLC that differ in the types of crystal lattices of their crystallites, the hardest is tetrahedral amorphous carbon. Young’s modulus of DLC may reach over 500 GPa, similar to that of natural diamond. The discovery of DLC was announced by Aisenberg and Chabot in 1971 [70]. The material has been since intensively used for hard coating protection of steel machine tools and has excellent tribological properties.

Freestanding DLC nanomembranes were used as ultrafast nanofilters of viscous liquids in [71]. They showed high hardness and at the same time resistance to organic solvents, similar to thin DLC films deposited on metal substrates.

##### Semiconductor Nanomembranes

Among the most often used semiconductor nanomembranes are those made of silicon [56]. Due to maturity of silicon microelectronic and microsystem fabrication technologies, processes are available that enable production of relatively low-cost and high-quality freestanding Si nanomembranes with arbitrary thickness and giant aspect ratios. Such membranes are used in a plethora of applications due to their favorable properties, which include high flexibility (convenient for foldable and stretchable electronics), good thermal properties (due to phonon confinement in structures with nanometric thickness), desirable electrical and optical performance due to electron confinement in the quasi-2D structure (convenient for microelectronic and optoelectronic devices), good dissolution and degradability over time in bodily fluids (convenient for implantable structures intended for biomedicine), etc.

The most frequently used technique for fabrication of high-quality freestanding single crystalline silicon nanomembranes is etching of commercially available silicon-on-insulator (SOI) wafers, first that of silicon in order to obtain the desired thickness of the thin top layer, then the sacrificial etching of the buried silica layer in order to completely remove the nanomembrane from the substrate. Layers with thickness as low as 2 nm have been thus fabricated, although typical values of SOI-obtained Si nanomembrane thickness fall in the range between 20 nm and 50 nm.

Other semiconductor materials can be formed into freestanding nanomembranes as well. Examples include molybdenum disulfide (MoS_2_) whose bandgap from indirect in bulk materials transforms to a direct one in nanomembrane form, germanium sulfide (GeS), germanium selenide (GeSe), perovskite Sr_2_Nb_3_O_10_, among others [72]. An often-used method to produce such nanomembranes is either chemical etching exfoliation (for instance, in aluminum arsenide/gallium arsenide sandwiches the AlAs layer is sacrificially etched in the course of exfoliation or, as another example, in aluminum gallium arsenide (AlGaAs)/gallium arsenide (GaAs) multilayers the AlGaAs layers are sacrificial.)

##### Freestanding Monatomic Sheets

The prototype monatomic material is graphene [73,74,75]. It is a crystalline allotrope of carbon in the form 2D hexagonal lattice consisting of a single layer of carbon atoms. Its mechanical strength is two orders of magnitude greater than that of steel. Graphene represents a zero-bandgap semiconductor, its valence band touching its conduction band, and shows plasmonic behavior. Atomically thin freestanding graphene membranes have been made, and they showed high mechanical robustness and chemical stability [76]. They can be made porous or non-porous. Their proposed applications include DNA sequencing, water filtering and purification and various sensing applications. 

Besides carbon, other chemical elements that form 2D allotropes include 2D silicon (silicene), 2D boron (borophene), 2D phosphorus (phosphorene or black phosphorus, also blue phosphorus) 2D germanium (germanene), 2D tin (stanene), 2D lead (plumbene) and 2D bismuth [45]. All of these 2D sheets have been experimentally produced by exfoliation technique, by physical vapor deposition, PVD or chemical vapor deposition, and CVD (including atomic layer deposition, ALD).

##### Freestanding Inorganic Monomolecular Sheets

The prototype monomolecular materials are MXenes [40], defined as compounds consisting of carbides, nitrides or carbonitrides of early transition metals (Ti, Cr, Sc, V, Nb, Zr, Hf, No or Ta). Their atoms form 2D monolayer sheets that are interconnected into laminar structures such that the manner of obtaining the sheets is to exfoliate them by chemical etching. They can be described as electrically conductive clays. There is a large number of 2D materials that belong to MXenes, with a wide range of different properties, with the most frequently encountered one among them being titanium carbide [77]. Other 2D compound materials include gallium arsenide, transition-metal dichalcogenides, sulfides, selenides and tellurides of tungsten, niobium, molybdenum or tantalum, aluminum carbide, cadmium selenide, and many more.

#### 2.3.2. Organic/Inorganic Hybrids

##### Interpenetrated Structures

Usually this term denotes structures made of polymers and reinforced or otherwise functionalized by inorganic nanofillers. As an example, in 2006 Vendamme et al. produced hybrid organic/inorganic nanomembranes using spin-coating. They fabricated freestanding, 35-nm-thick membranes with lateral dimensions of several centimeters consisting of polyacrylate interpenetrated with zirconia (ZrO_2_) [51]. In 2007 the same research team fabricated another type of large interpenetrated organic/inorganic membranes, this time a few tens of nanometers thick, of elastomeric polyacrylate networks interpenetrated with silicon dioxide [78]. Polymer membranes to which reinforcing nano-building blocks were added could also be classified to this group, a prime example being nanomembranes reinforced with single- or multiple-walled carbon nanotubes (CNT.)

##### Metal-Organic Frameworks

Metal-organic frameworks (MOF) represent 1D, 2D or 3D nanocrystalline compounds, usually with a highly porous structure, which consist of metal ions or ion clusters and organic molecules [79,80]. Their tailorable porosity, combined with adsorbability, makes MOF nanomembranes convenient for different highly selective sensing applications, as well as for their use as efficient molecular sieves [81].

#### 2.3.3. Organic Nanomembranes

Completely organic freestanding nanomembranes that are in entirety composed of one or more organic materials represent a huge class of the existing freestanding nanomembranes. There is a vast number of organic compounds and their combinations that could be used. 

Organic materials are defined as carbon compounds. That means that they do not include pure carbon in any of its allotropic forms. However, this classification also excludes the simplest compounds of carbon, like oxides of carbon, carbonates, carbides, and cyanides. All other carbon compounds belong to organic substances. This does not mean that any organic compound can be used to make nanomembranes—some of these compounds are gaseous or liquid, while others simply cannot be formed into membranous structures. It appears that the best suited organic compounds for production of freestanding nanomembranes are many of those with macromolecular/polymeric structure. They are characterized by large molecules, sometimes very large. These include polysaccharides [19], synthetic lipids [82], synthetic polymers [83], proteins [84], DNA [85] and RNA [86]-based membranes.

Most macromolecular nanomembranes are characterized by some common traits. They are usually highly sensitive to elevated temperatures and actually retain their useful properties in a narrow temperature range, are attacked and often dissolved by organic solvents and sometimes are even endangered by increased humidity. Their mechanical properties tend to be impaired with a decrease of membrane thickness. Most of them have a low Young’s modulus. They usually start to creep and are permanently plastically deformed under permanent stress. Probably the first produced organic nanomembrane was made in 1907 by Bechhold from pyroxylin (nitrocellulose, collodion) [87].

Here we consider some exemplary types of macromolecular nanomembranes, those consisting of synthetic polymers. Depending on their composition, they may be classified into single-compound (pure) [88], and blended polymer (copolymer) [89] nanomembranes.

##### Single-Compound (Pure) Organic Nanomembranes

As an example, here we quote a small number of polymers used to date to fabricate macromolecular nanomembranes. They include epoxy resins [88], polysulfone [90], polyethersulfone [7], polyacrylate [91], polycarbonate [90], polystyrene [92], nylon [93], cellulose [94], nitrocellulose [87], generally polysaccharides [19], polyamide [90], polyimide [95], polydopamine [96], polypropylene [90], polyurethane [19], poly(methyl methacrylate) (PMMA) [97], polyvinylchloride (PVC) [90], polyester [98], polytetrafluoroethylene (PTFE, Teflon) [90], poly(vinylidene fluoride) [90], poly(lactic acid) [99], polyacrylonitrile (PAN) [90], polydimethylsiloxane (PDMS) [90] and many more. There is a host of convenient macromolecules besides those listed here and their systematic classification falls by far outside the scope of this work.

##### Polymer-Composite (Copolymer) Organic Nanomembranes

Probably the most often used polymer-composite blend is block copolymers [89], simultaneously made from two or more monomers and consisting of homogeneous blocks of pure polymers whose number is identical to that of the monomers used for polymerization. Numerous combinations of the above-listed polymers can be also used to fabricate copolymers, as well as many macromolecules not even mentioned here. The number of possible combinations is literally endless. More details on block copolymers and their ordered self-assembly can be found later in this text, in Section 3.2.

##### Carbon Nanomembranes

Carbon nanomembranes (CNM) [100] actually represent freestanding monomolecular sheets of cross-linked carbon precursors, fabricated by self-assembly. Thus, their name is somewhat misleading, since they are not composed solely of carbon atoms as one may assume, but are based on carbon instead. Thus, they are a separate group within the wider class of organic nanomembranes. They can, however, be converted to freestanding graphene sheets, for instance using pyrolysis. CNM are about 1 nm thick (a single molecular layer), and despite this they show remarkable mechanical strength. They can be produced as continuous surfaces or they can be built with a system of pores. Their precursors can be a variety of organic molecules, including but not limited to, polyaromatic thiols—oligophenyls and condensed polycyclic hydrocarbons. Recently, terphenylthiol monomolecular CNM nanomembranes with subnanometer pores acting as water channels were used for ion exclusion filtering and separation [101].

The production of CNM includes self-assembly of organic precursors into ordered monolayers of molecules on a solid surface, their subsequent cross-linking by forming covalent bonds between neighboring molecules using electrons, electromagnetic radiation or ions, and finally their release from the surface as free-standing or free-floating monolayer nanomembranes. The CNM unite extremely low thickness of graphene with simple production and easy and versatile surface functionalization, some examples of which include linking with fluorescent materials, dies, target-specific ligands or biomolecules. There are some excellent review papers of CNM, including [47] and [100]. They cover the fabrication of carbon nanomembranes, their functionalization and applications.

#### 2.3.4. Model Lipid Bilayers 

Model lipid bilayers are actually synthetic lipid bilayer nanomembranes. Strictly speaking, they could be classified into the previously described wide group of organic nanomembranes. However, since they represent replicas (and in some cases even upgrades) of the living nanomembranes, we decided that they merit a class of their own, belonging more to synthetic biology than to general organic chemistry.

Historically, the first model lipid bilayers were synthesized in 1962 [102], and were at first known as the so-called “black lipid membranes” and also as “painted bilayers”. They were intended as scaffolds to study the membrane processes in vitro, to facilitate the analysis of the transmembrane mechanisms and the function of ion channels. 

Many different model lipid bilayers were subsequently fabricated, and they are being developed at this very moment. A review of methods and approaches to synthesis of artificial lipid bilayer membranes has been written by Siontorou et al. [103].

Today functionalized model lipid bilayers are built, among other purposes, for experiments with artificial cells in synthetic biology (the ultimate research subject in biomimetics—the fabrication of biochemical “artificial life” i.e., “synthetic biogenesis.”)

## 3. Fabrication

### 3.1. General Strategies

In this subsection we present the three main generic approaches to the fabrication of freestanding nanosheets/nanomembranes. This classification is based on the prevailing strategies used in micro- and nanomanufacturing. Besides the well-known top-down approach, used in the semiconductor industry and in microsystem fabrication, and the bottom-up approach, used for chemical synthesis and most often applying various self-assembly techniques, here we quote the third method, exfoliation of nanometer-thick sheets from starting macrostructures, which may be obtained in a variety of ways. As far as the starting substrates go, they may be solid or liquid. A summative table is given below (Table 2).

In most situations, after the fabrication procedure, nanomembranes will have to be either completely freed from the surrounding liquid solution by direct removal from it, or in some cases by replacing the original liquid with another one. Liquid interchange represents an intermediate step before extracting the membrane. In that case, one replaces a liquid with a higher surface tension that could damage or destroy the membrane during extraction with one having a lower surface tension.

#### 3.1.1. Solid Substrates and Etching of Sacrificial Structures

This is a method with its roots in microelectromechanical system (MEMS) technologies, i.e., it belongs to the top-down procedures. In short, one first chooses some kind of material (often silicon) as a substrate. A layer to be selectively etched and thus removed (“sacrificed”) at a later stage may then be deposited on the substrate. An ultrathin (thickness below 100 nm) layer of material to become a nanomembrane is deposited over the sacrificial layer. Some means of etching that selectively removes the sacrificial layer material is then applied. After this process, the nanomembrane remains free where the sacrificial layer was. If the etchant was a liquid, the nanomembrane remains freely floating in the etchant solution or, alternatively, suspended on its edges connected to the solid substrate. If a non-liquid etching procedure was applied, then the latter part of the procedure is still valid and one is again left with a freely suspended ultrathin structure.

A variant of this approach is that the substrate, made of single crystalline silicon, is directly etched (for instance, through an opening in a previously deposited photolithographic mask) using anisotropic bulk micromachining. Etching is applied through the mask opening and finished when the nanomembrane layer is reached, since etchant does not react with it. The remaining part of the substrate serves as the edge support for the suspended freestanding nanomembrane. In this manner, the substrate performs the dual role of being simultaneously the support and the sacrificial layer.

#### 3.1.2. Fabrication on a Liquid-Air Interface

Among the generic methods for nanomembrane fabrication, an important position belongs to structures fabricated without a solid substrate. Most of the self-assembly (bottom-up) methods and generally chemical synthesis of ultrathin free-floating films may occur at liquid-air interfaces (the surface of liquids). Basically, the method consists of synthesizing or depositing a free-floating film on the surface of a liquid. Many ultrathin film deposition methods used with solid substrates (top-down) are applicable here as well. The difference is that here one does not have to produce a sacrificial layer, and instead finishes with the nanomembrane already floating on the surface of the solution. The approach is especially useful for soft organic membranes. The method has even proven itself convenient for high-quality nanocrystalline nanomembranes [104].

#### 3.1.3. Exfoliation

The third generic procedure for nanomembrane fabrication is direct detachment of monatomic/monomolecular or multilayer films from a bulk material. It is used if the starting material already has a laminar structure (i.e., consists of nanosheets of material to become nanomembranes or “nanoflakes”. Such is the case with many materials used to produce monolayers, e.g., graphene, various types of MXenes, etc. Single or multiple layers may be detached from the structure using the “Scotch tape” method, i.e., by literally sticking a piece of adhesive tape to the laminar structure with weak interlayer bonds and simply pulling it off, after which flakes of monatomic/monomolecular nanomaterial remain attached to the tape [75,105]. In other words, this kind of exfoliation is done in a purely mechanical way. This method has been successfully applied to obtain even relatively large graphene monolayers (size on the order of a millimeter). It was the chosen method when fabricating the first graphene monolayers. 

Another approach to exfoliation is applied if the bond between layers is strong enough to render mechanical cleavage useless. In such situations, one uses a liquid etchant to remove the bonds between the neighboring monolayers, leaving them freely floating in the etchant solution. This is the approach used to obtain MXenes [106]. Other exfoliation methods include the electrochemical approach [107], sonication using ultrasound in some kind of fluid (e.g., ionic liquid, mixture of two immiscible liquids) [108], cleaving by a sharp edge, shearing by mixers, etc.

### 3.2. Nanomembrane Production Methods

At the beginning of this subsection we would like to describe a distinction between additive/subtractive manufacturing and top-down/bottom-up approach, which denote completely different processes. The additive/subtractive fabrication regards what is done with the membrane material (additive—new material is added to the membrane; subtractive—material is removed from it, typically to obtain a pore). The top-down/bottom-up approach regards how addition/subtraction is done (top-down refers to standard planar, microelectronic, MEMS and NEMS batch processing, while bottom-up is actually self-assembly). Both top-down and bottom-up approaches can be used to add or remove material.

#### 3.2.1. Top-Down Approach: Thin Film Technologies

A majority of ultrathin film deposition techniques used in microelectronics and microsystem (MEMS) technologies can be used in the production of biomimetic nanomembranes as well, combined with the sacrificial layer etching technique. An excellent source of information on different microsystem techniques of deposition in general can be found in the seminal work of Madou [109]. Previously, we reviewed different techniques applicable for nanomembrane fabrication in [110], so in this work we cover the field only briefly, accentuating the new developments that have become prominent in recent years. For a more in-depth approach, although it is necessarily an older report, we direct the readers to the mentioned review.

The top-down deposition techniques can be divided into two main groups: physical methods and chemical methods. We only list here the main procedures. Some procedures are present in both lists, because they may include chemical processes, but do not have to.

Physical methods for depositing nanomembranes in a controlled way include radiofrequent sputtering, evaporation (e.g., thermal evaporation and laser-assisted evaporation), physical vapor deposition (PVD), epitaxial growth (homoepitaxy or heteroepitaxy), spin coating (aka spinning or spin casting—only if no chemical changes and no self-organization occur when depositing thin films in this manner), drop-coating, dip-coating, electrospray deposition, molecular beam epitaxy, ion beam deposition, electron beam (e-beam) deposition, atomic layer deposition or molecular layer deposition, cathodic arc deposition, pulsed laser deposition, etc.

Chemical methods encompass chemical vapor deposition (CVD), plasma enhanced CVD, again atomic layer deposition (ALD), plating (including electroplating), sol–gel method (chemical bath deposition), spin coating (if a chemical process like polymerization occurs during the process), again molecular beam epitaxy, etc. For exhaustive details on the mentioned methods, the reader is directed to the above-referenced work of Marc Madou.

#### 3.2.2. Bottom-Up Approach: Self-Assembly Methods

Self-assembly is a natural process of structures organizing themselves on their own into larger units, as defined by the properties of their smallest constituents (their geometry, chemical and physical properties). It is an ubiquitous process, and its examples can be encountered in a vast range of dimensions and systems, from atoms and molecules through viruses to galaxies and metagalaxies [111]. Thus, it is truly omnipresent and not only multiscale, but rather an “all-scales” process.

Self-assembly can be defined as a process in which the constitutive parts of a system reach an ordered spatial distribution within the boundaries of the system through self-organization. A review of self-assembly methods and approaches used for freestanding nanomembranes can be found in [112]. Here we mention some of the most prominent techniques used in self-assembly.

##### Langmuir-Blodget Method

The Langmuir-Blodget (LB) technique [113] is a biomimetic self-assembly procedure. It uses molecules possessing a lipophilic (hydrophobic) “tail” and a hydrophilic “head” (amphiphiles or surfactants—surface-acting agents); examples include fatty acids, phospholipids, and glycolipids, but also various polymers like polyimides, as well as some inorganic nanoparticles. When placed at an air–water interface, a monomolecular film is formed (a Langmuir monolayer), since the amphiphilic molecules orient themselves to minimize free energy. Thus, the formed monomolecular surface layers are insoluble in water.

##### Layer-by-layer (LbL) Self-Assembly

Layer-by-layer deposition also belongs to self-assembly methods [13]. It uses adsorption of alternating macromolecular layers, each with an opposite electric charge with respect to the previous. It proceeds in the following manner: a dilute solution of a cationic (+ charge) material is brought to a substrate and is there adsorbed in a single monomolecular layer whose exact thickness will be determined by the particular molecules used for the monolayer adsorption, but as a rule of thumb it will be on the order of a nanometer. In the next step, after rinsing and drying, the substrate covered with cation is placed into a dilute solution of anions (− charge), and thus a new monolayer is adsorbed on the previous one, and the wafer is again rinsed and dried. Now the process may be repeated a desired number of times. When designing a multilayer, arbitrary chosen materials can be used under the condition that their charges are alternating. The method gives multilayers with a thickness from about 5 nm to over 500 nm. Due to its simplicity, the method has been called the “molecular beaker epitaxy”. This is a popular method, often used to produce high-quality giant aspect ratio nanomembranes.

##### Block Copolymer Self-Assembly

A copolymer is a polymer produced by simultaneous polymerization of two or more starting monomers. A block copolymer is a kind of copolymer in which there are two or more distinct kinds of blocks that consist of a single pure copolymer (homopolymer) produced from one monomer and are chemically different and even immiscible. These blocks are covalently bonded among themselves. Block copolymers in solution naturally tend to self-organize in various shapes. To date, more than 20 aggregate shapes have been recognized, including spherical micelles, lamellae, rods, tubules, onion-shaped forms, egg-shaped forms, tubules, etc. An excellent tutorial on block copolymer self-assembly has been published by Mai and Eisenberg [114]. Self-assembly of block copolymers has gained distinct popularity for fabricating complex dielectric nanostructures for optical, photonic crystal and metamaterial applications [29]. Its application in the fabrication of various types of freestanding nanomembrane has been extensive. One of the useful traits of block copolymer self-assembly is that it generates ordered patterns that can be useful for membrane functionalization, while at the same time producing the membrane and performing its functionalization. Examples include self-supported perforated polymer nanomembranes intended for protein separation [83] and UV-polymerized nanomembranes of phospholipid and copolymer fabricated by Langmuir-Blodgett method at the air–water interface [115].

##### Sol-Gel Process

Sol-gel technology is a deposition procedure in which sol (a colloidal solution) deposited on a substrate gradually sets into a gel (semi-solid colloidal suspension) consisting of a continuous integrated solid network of nanoparticles or/and polymer in liquid. The excess liquid is removed e.g., by centrifugation. This step is followed by drying and possibly heat treatment (firing). Under some conditions, nanoparticles will self-assemble while setting. This technology has been very popular for the fabrication of different types of freestanding nanomembranes, including interpenetrated polymer-zirconium oxide membranes with giant aspect ratio [51]. The technique has been used for the fabrication of sol–gel on-polymer processed indium zinc oxide intended for wearable soft electronics [116].

##### Dip-Coating

**Figure 2:**
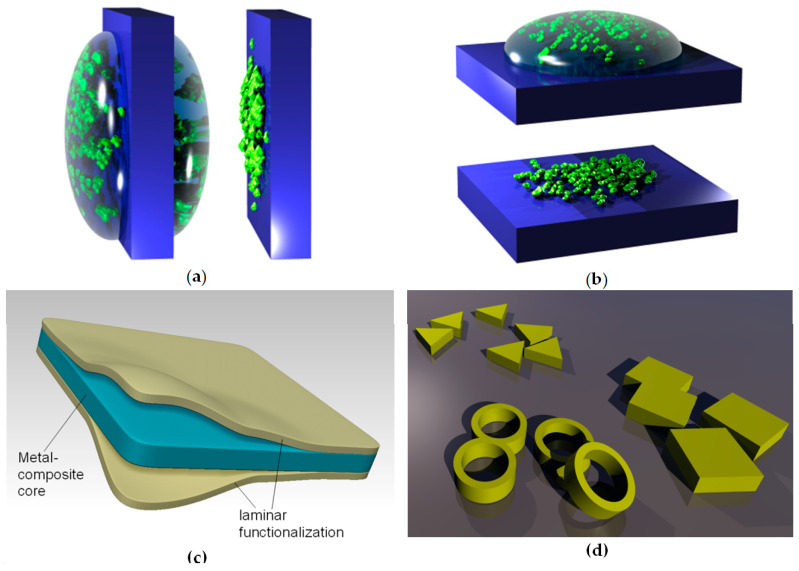
Illustration of dip-coating and drop-coating techniques: (**a**) Dip-coating; left: before drying; right: after self-assembly of nanoparticles and drying. (**b**) Drop-coating; top: before drying; bottom: after self-assembly of nanoparticles and drying. (**c**) An example of functionalization of synthetic nanomembrane by lamination. A larger number of layers can be used, as well as different materials for any of them. Authors’ own work from [52]. (**d**) Some different shapes of nanoparticles that can be used as nanofillers (a small and arbitrarily chosen part of what became known as the “nanotechnological zoo”).

Dip-coating is a method of depositing thin films where an object to be coated is dipped (immersed) in the solution containing the material to be deposited or, alternatively, a suspension of its nanoparticles. The object is left for some time in the liquid and then it is withdrawn from it. During the emersion, a layer of liquid remains attached to its surface. The object is held for some time for the excess liquid to flow and drop from it. After drying or evaporation, the nanoparticles remain attached to the surface in the form of a film, whose thickness can be tailored by the amount of liquid and the filler material dissolved or suspended in it. Care must be taken that the liquid uniformly covers the surface. The procedure is convenient for sol–gel and hydrogel deposition, as well as for deposition of self-assembled monolayers (although self-assembly may be or may be not present, depending on the process materials and parameters). Dip-coating is convenient for large-scale industrial production, since the object may be continuously movable (e.g., a tape). Figure 2a illustrates the dip-coating process.

##### Drop-Coating

**Figure 3:**
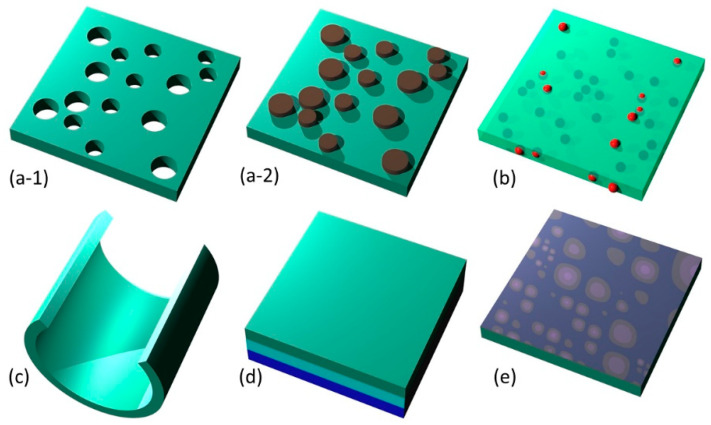
Summary of main methods of nanomembrane functionalization. (**a**) nanopatterning: (**a-1**) subtractive patterning; (**a-2**) additive patterning; (**b**) nanofillers; (**c**) rolled (strain-engineered) nanomembrane; (**d**) lamination (multilayering); (**e**) surface activation.

Drop-coating is a method of depositing thin films where drops of the solution or suspension containing the material to be deposited are sprinkled on the surface of the object to be coated in accurately controlled quantity. The object is left until all of the liquid dries through drying or evaporation, leaving a solid film or nanoparticles attached to the surface. In this case, like in dip-coating, the thickness of the film can be tailored by the amount of liquid and the filler material dissolved or suspended in it. Once again, care must be taken that the liquid uniformly covers the surface. This procedure is convenient for sol–gel and hydrogel deposition, as well as for deposition of self-assembled monolayers. Figure 3b illustrates the drop-coating process.

## 4. Functionalization

Similarly to their biological counterparts, the role of synthetic nanomembranes would be more or less purely mechanical and protective if there were no additional functionalities. Most of the roles performed by biological nanomembranes are due to built-in protein structures that ensure additional functions. A very similar situation is encountered in synthetic structures. The difference is that structures enabling biological functionalities are much more sophisticated than the artificial ones, but for the price that they resort to a much smaller toolbox, in the sense of chemical composition and the available choice of materials, operating temperature and humidity ranges and available functionalities. With artificial structures the methods are much more crude and imperfect, but the number of possible ways to perform functionalization is much larger, and the material choice is wider, as is the pure range of functionalities. The latter include many options and paths not encountered in nature. One is free to avoid the limitations of protein chemistry and reach for a much larger toolbox to incorporate in a biomimetic way many novel non-biological properties, including plasmonic, magnetic, electric and optical ones. In 2010, the authors published a review of possible approaches to multifunctionalization of nanomembranes [117]. Here we give just a brief overview of the methods and approaches, concentrating to the more recent findings published in the meantime and to the methods that were skipped in [117].

### 4.1. Five Basic Methods of Functionalization

#### 4.1.1. Lamination/Multilayering

Lamination is a method of functionalization by making multilayered structures, each layer contributing its own properties and functions. It is the method often used by nature in biological membranes. It is also the simplest method of functionalization, but is very versatile nevertheless. Any of the fabrication methods listed in Section 2.2 can be used. The number of layers can be arbitrary, as can be the added functionalities.

An illustration of the use of lamination to functionalize biomimetic membranes is shown in Figure 3c. It shows a metal composite nanomembrane in the middle, with two external layers enveloping it from both sides. These external layers can consist for instance from a biocompatible material, thus ensuring a possibility to use the structure for biomedical and life science application. 

#### 4.1.2. Nanofillers

One method to functionalize a nanomembrane at the fabrication stage is to incorporate nanoparticles, nanofibers, etc., into its body (the host) [110,117]. One must take care during design not to compromise the existing desirable properties of the nanomembrane. 

For instance, a nanomembrane structure may be mechanically reinforced by incorporating carbon nanotubes. One could incorporate one type of nanofillers only or several types simultaneously, each with its own functionality, thus obtaining multifunctionality. Examples include incorporation of nanofillers with biological or chemical functions, catalytic (including photocatalytic), magnetic, light-emitting, plasmonic, piezoelectric, etc. A nanotechnological “zoo” showing some possible nanofiller geometries is presented in Figure 3d. Naturally, the number, the kinds, types and materials of available nanofillers vastly surpasses what is shown in Figure 3d for illustrative purposes only.

#### 4.1.3. Nanopatterning

Functionalization of a nanomembrane may be done by defining a pattern on it by adding material (additive approach) or by removing it (subtractive approach)—the fabrication of nano-topographies. Bearing in mind their minute thickness, subtractive patterning of nanomembranes is usually equal to pore making. The patterns may be ordered (arrayed) or disordered. The same or different pattern motifs may periodically repeat in one or both directions across the membrane surface. Additive patterning may be done in a single layer or in multiple layers.

##### Top-Down Approach to Additive/Subtractive Patterning

While still on the surface of the sacrificial layer, the nanomembrane may be processed using any of the microelectronic/microsystem (MEMS) or nanoelectronic/nanosystem techniques [118]. The standard photolithographic procedures may be used to define patterns for both additive and subtractive patterning, but only if the features are larger than 130 nm. The smallest details are determined by Abbe diffraction limit, according to which the resolution achievable by light is the operating wavelength divided by about 2.8 (depending on the optical equipment used). Since the standard photolithographic light sources operate at 365 nm, this gives us the detail size given above. The same is valid for direct laser writing techniques that may be used to delineate motifs in the photoresist mask, or even to directly process the membrane material. For the definition of details of the order of a nanometer, one has to resort to more recently developed nanolithography methods [118,119,120], like electron beam lithography, focused ion beam lithography, proton beam lithography, neutral particle lithography, extreme UV lithography, X-ray lithography, synchrotron radiation lithography, magnetolithography, nanoplasmonic lithography, liquid immersion lithography, quantum optical lithography, stencil lithography, nanosphere lithography, etc.

One of the favorite tools for fabrication of nanometric details is nanoimprint lithography [121]. Near-field optical methods can be used to obtain nanometric details, like superlenses based on metamaterials. Another method is scanning probe nanolithography (which includes the dip-pen method, direct writing by scanning microcantilever, thermal and thermochemical scanning probe nanolithography, scanning probe lithography, local oxidation nanolithography, etc.).

Most of the nanolithographic methods listed can be also used for subtractive patterning, i.e., pore forming. Some of them have been successfully used directly on freestanding nanomembranes, for instance, focused ion beam [122].

Material for additive patterning may be deposited through the mask openings, or removed by selective etching when forming pores. Most of the standard deposition techniques can be used for additive patterning, even at nanodimensions. These include (in no specific order) sputtering, electrochemical deposition, chemical deposition, CVD, ALD, PVD, local epitaxy (including molecular beam epitaxy), thermal evaporation, laser evaporation, vacuum arc deposition, electric arc deposition, etc.

##### Bottom-Up Approach to Additive/Subtractive Patterning

Nanometric and even subnanometric features may be defined by self-assembly techniques [123] using various supramolecular chemistry approaches and starting from various precursor materials [124], including both additive and subtractive patterns. Regarding fabrication of nanopores, a very convenient pathway to it are those processes and materials for fabrication of nanomembranes which intrinsically result in fabrication of nanopores in ultrathin sheets, for instance by using supramolecular self-assembly. In this way, regular nanoporous networks were fabricated, with the nanopore diameters ranging from 1 nm to over 10 nm [125]. Various supramolecular pore shapes can be produced, including circular, hexagonal and square, as well as various dimensions in the nanometric range. The Langmuir-Blodget method can be useful for additive patterning, and layer-by-layer for both additive and subtractive. Due to the quality and a large variety of available geometries, a prominent place among bottom-up nanomembrane patterning methods belongs to block-copolymer self-assembly. 

#### 4.1.4. 3D Sculpting

Nanomembranes are characterized by high tailorability and customizability. They can adjust their shape to whatever body they are transferred to. Despite their minuscule thickness, even when freestanding, they can generally be sculpted into various 3D shapes that are surprisingly robust. Such surface formations include waves [78], pyramids [126], rolling into tubules [123,127], nano-origami [128,129] and nano-kirigami [130,131], spirals, curved sheets [132], as well as other structures [123].

One can use built-in stresses in nanomembranes to fabricate various 3D shapes (tubules, spirals, Swiss rolls, helicoids, etc. [65,66], or, alternatively, the sacrificial layer may be sculpted before depositing nanomembrane layer; after sacrificial etching, the shapes remain permanently sculpted in the freestanding structure [126].

A technique of choice to induce folding of nanomembranes into 3D shapes is strain engineering. To this purpose, one induces spatially distributed strain into a nanomembrane or a nanoribbon, which may be done by built-in structuring or externally in a mechanical way. Many physical properties are changed in strain-deformed nanomembranes, and they sometimes effectively behave as if they were fabricated in a completely new material. Examples of strain engineering in the fabrication of nanomembranes include, e.g., [133,134].

#### 4.1.5. Surface Activation

Surface activation or surface modification represents the application of some external means to a part of or the whole surface of the nanomembrane to modify the behavior of its interfacial parts that interact with the environment [135]. The implemented changes may be located strictly at the interface with the environment or may spread throughout the volume of the nanomembrane.

The activation procedures may include chemical, electrochemical, photochemical, etc., treatment [136]; they may involve irradiation with ionizing electromagnetic waves, including extreme UV, X-ray and gamma ray [137]; or bombardment with nuclear particles (alpha and beta particles, neutrons and protons, accelerator particles). Illumination by non-ionizing electromagnetic waves may include radiofrequent or microwave radiation, infrared, visible or near-UV rays. Last but not least, plasma processing is a widespread method to activate membrane surfaces [138].

Surface activation may involve breaking surface bonds or generating new ones, removal or altering of the boundary atoms/molecules, creation or destruction of polar groups, etc. This may introduce dramatic changes in the nanomembrane parameters and modify them even for several orders of magnitude.

Activation processing may affect practically all of the membrane parameters. For instance, the diffusive properties of a biomimetic nanomembrane may be modified by adding a specific chemical agent. An example is the work of Sharma et al., who applied capsaicin to the membrane surface in order to change its interaction with certain molecules [139]. Chemical activation of diamond and silicon surfaces for biosensing of proteins was investigated in [140].

As a visual summary, Figure 3 shows the five main methods of nanomembrane functionalization. These include nanopatterning (subtractive, a-1, and additive, a-2), nanofillers (b), 3D-sculpted nanomembrane (rolled-up) (c), lamination (d), and activated surface (e).

### 4.2. Structures for Nanomembrane Multifunctionalization

#### 4.2.1. Synthetic Ion Channels

Ion channels in biological cell membranes are ionophoric transmembrane protein receptors that selectively facilitate the transport of ions in the direction of lower concentration and charge, i.e., down the electrochemical gradient. An ion itself could not pass the hydrophobic lipid bilayer wall since it requires the presence of an aqueous environment to retain its ionic structure and is destabilized without it. An ion channel represents a gateable pore (i.e., a structure that can be opened/closed by some of the gate mechanisms) that replaces interaction between the ion and water with an equivalent interaction between the ion and the channel, thus ensuring a decrease in the barrier energy for its transport.

There are many kinds of synthetic ion channels [141,142]. Probably the oldest is gramicidin, a semi-synthetic ion channel that acts as an ionophoric substance, first obtained in 1939 by René Dubos and still used nowadays as an antibiotic. It has a helical peptide structure, with a pore inside. When built into a lipid bilayer, it ensures a high-permittivity path through it, acting as a channel for light metal ions like sodium (Na^+^) or potassium (K^+^). In 1977 Kennedy et al. synthesized four different peptides and reported their properties as synthetic ion channels in vitro after incorporating them in their black lipid membranes (model lipid bilayers) [143]. The first fully synthetically produced ion channel was tetra-substituted β-cyclodextrin [144], which was reported as early as in 1982.

**Figure 4:**
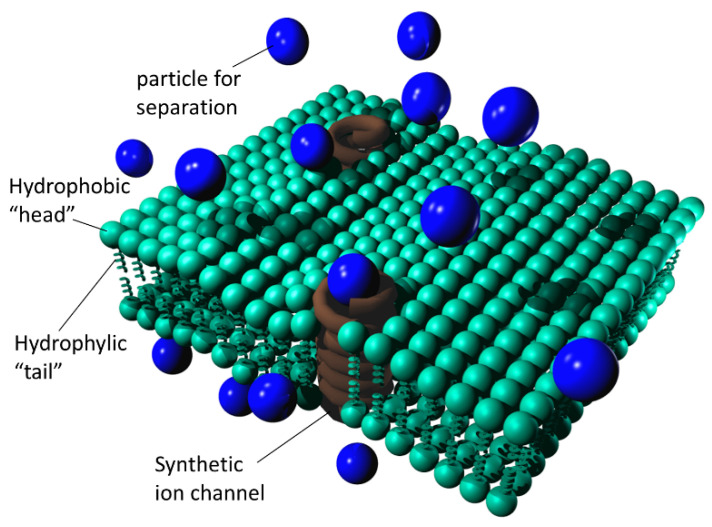
Simplified depiction of a freestanding lipid bilayer nanomembrane with built-in synthetic ion channels; blue spheres are particles to be separated, while brown helices represent synthetic ion channels.

Figure 4 shows a simplified depiction of a freestanding lipid bilayer nanomembrane with built-in synthetic ion channels (shown in a simplified manner as spirals).

Artificial ion channels may be based on cyclodextrin (the first synthesized ion channel), calixarenes, macrocycles (including peptide macrocycles), G-quadruplex, p7 viroporin, etc. 

Their opening or closing (gating) may be controlled by different mechanisms [142]. The gating mechanisms include

- Voltage gating (ionic switching) [145]
- Chemical gating (ligand gating) [146]
- Light (optical) gating [147]
- Mechano-sensitive gating [148]

#### 4.2.2. Synthetic Ion Pumps

Similarly to ion channels, the ion pumps are ionophoric transmembrane protein receptors that enable selective and controlled ion transport through lipid bilayer, but, contrary to ion channels, they ensure transport against the electrochemical gradient, i.e., in the counter-direction relative to diffusion. Their function is perpetrated by the fluctuations of the external field.

The synthesis of ion pumps is much more complex than that of the ion channels. Synthetic ion pumps were reported in [149]. They fabricated an asymmetric nanostructure with a narrowing channel (a “nanopump”), so that it functions as a kind of electrochemical ratchet. In this manner, they ensured a net flow of potassium ions against the direction of the electrochemical potential, rectifying the ion current. The rectification amount is dependent on the angle of the conical nanopore, as well as on its side length. The concept actually represented a scaled down version of a previously proposed microsystem that performed the same function on micrometer-sized particles [150]. The principle of conical pore-based synthetic nanopumps proposed in 2002 is still used today [151], and the production of nanocone ion rectifier pumps remains a daunting task.

#### 4.2.3. Artificial Water Channels

Aquaporins or water channels are integral channel proteins forming pores in cellular membranes. Their main role is to facilitate selective transmembrane transport of water, which proceeds in addition to the osmotic transport directly through the lipid bilayer. They also enable, in a smaller amount, the transport of some smaller neutral solutes (urea for instance), as well as gases (carbon dioxide, ammonia). They consist of six alpha helices having amino and carboxylic terminals within the cytoplasmic part, while the inner wall is padded by asparagine-proline-alanine complex. They have an hourglass structure with the diameter at the narrowest part of 0.3 nm. Water molecules in it form a “water wire”, i.e., a single file molecule array. A cluster of amino acids called the aromatic/arginine filter selectively binds to water molecules and ensures their passage while blocking other molecules that do not bind to it. The efficiency of their transport is up to 10^9^ molecules per second per a single aquaporin channel. There is several various types of aquaporins in both plant and animal cell membranes.

Peter Agre was awarded the 2003 Nobel Prize in Chemistry for the discovery of aquaporins. Natural biological aquaporins are built into separating membranes and used in water separation at an industrial scale. However, they are expensive, have low stability and pose constraints for both fabrication of separators and the range of their operating conditions. This was the reason synthetic alternatives were sought for. The synthetic structures serving a function analogous to natural aquaporins are called artificial water channels [152,153].

Carbon nanotubes (CNT) with an inner diameter of about 0.9 nm, which allows the formation of water wires, have been proposed as artificial water channels for incorporation into nanomembranes [154]. For this purpose, short CNT (5–20 nm) with diameters 0.9–20 nm are used. They easily self-incorporate and self-align in lipid bilayer membranes.

Other artificial water channels can be based on single molecules or supramolecular assemblies. The first synthetic water channel was reported in [155], and was based on imidazole quartets. 

Single molecular channels include the PAP1 pillar[5]arenes, as proposed by Hu et al. [156]. PAP1 molecules are impermeable to protons, but show no selectivity against cations. The next solution included PAP2 pillar[5]arenes incorporating peptidic poly phenylalanine arms. Their water permeability is comparable to that of aquaporins. In addition to that, they are permeable to amino-acids, but show a poor ionic selectivity.

Self-assembled supramolecular channels include tubular imidazole I-quartets as described in [155,157]. Other molecular assemblies used for artificial water channels include peptide-appended hybrid[4]arene (PAH[4]) [153], aquafoldamers, reverse osmosis (RO) membranes and double helical water T-channels [157].

Monolayer carbon nanomembranes based on terphenylthiol with subnanometric pores have been used as simple artificial water channels in [101]. A virtue of this solution is its simplicity, while such structures ensure extremely rapid passing of water (due to the shortness of the path the molecules have to pass) and at the same time show an outstanding rejection of undesired ions.

#### 4.2.4. Artificial Nuclear Pore Complexes

Nuclear pores are protein-based pores built into the nanomembranous envelope of the nucleus of a biological cell [158]. A nuclear pore is a part of the cell’s massive and highly complex nuclear pore complex (NPC), which serves as a scaffold for the translocation passageway. The role of an NPC is to mediate transport of macromolecules between the cell cytoplasm and the nucleus, but also to take part in genome organization and transcription processes. A nuclear pore complex consists of protein-based structures called nucleoporins (nups). There are 34 different nucleoporin proteins in each nuclear pore complex, containing about a thousand different proteins as their building blocks.

Biological nuclear pores are the largest pores in the cell. In vertebrate animals, the whole nuclear pore complex has a diameter of about 120 nm, while in other organisms it is smaller. The diameter of the nuclear pore channel is about 40 nm and its depth is about 45 nm.

The transport through the core of the NPC consists of export from the nucleus into the cytoplasm (this includes ribosomal proteins and RNA) and import from the cytoplasm to the nucleus (this includes proteins such as lamins and DNA polymerase, signaling molecules, lipids and carbohydrates) against the concentration gradient. Smaller molecules are transported through a nuclear pore by diffusion, while the larger ones are translocated only with the help of specific signal sequences based on karyopherin transport factors.

Jovanović-Talisman et al. succeeded in synthesizing artificial nuclear pores [159] in polycarbonate nanomembranes, consisting only of a passageway and its lining composed of scaffold-anchor phenylalanine-glycine nucleoporins (FG nups), which represent a binding structure for transport factors. Their artificial NPCs demonstrated nanoselective filtering and allowed passing of the transport factors and their cargo complexes that bind FG nups, while they simultaneously inhibited the transport of the proteins that did not bind these nucleoporins.

A large progress in understanding nuclear pore complexes has been achieved in recent times [160]. Still, many factors governing the behavior of biological NPCs and the role of several its sophisticated structural parts are yet to be understood, while the synthetic NPCs produced to date remain necessarily simplified.

#### 4.2.5. Artificial Organic Nanotubes

There are numerous synthetic structures, both organic and inorganic, in the form of tubes with nanometric dimensions that could be convenient for the use as ion channels. Generally, nanotubes can be made from basically the same toolbox as nanomembranes. Thus, inorganic nanotubes can be made of pure chemical elements (carbon, boron, silicon), inorganic compounds (tungsten sulfide, titania—titanium dioxide, gallium nitride, boron nitride, etc.) or their mixtures (including BCN, the composite of boron, carbon and nitrogen atoms in approximately same amounts).

One can introduce a classification of organic nanotubes in a similar fashion as we did for the inorganic ones. Organic nanotubes can be made from different macromolecules (examples being peptides, polysaccharides, block copolymers, macrocycles, artificial amphiphiles, oligophenylacetylenes, metal-organic polymers, etc.) or their mixtures (e.g., cyclic peptides containing beta-amino acids), but also from DNA molecules. A comprehensive review on self-assembled organic nanotubes can be found in [161]. Another approach is to produce hybrid organic-inorganic nanotubes, for instance by combining the above-mentioned materials with inorganic nanofiller as structural reinforcing agents.

Artificial inorganic nanotubes were, for instance, reported by Li et al. in [162]. They applied pyrolysis to lamellar mesostructures made of mixture of tungsten disulfide with surfactant. Thus, the layered sheets rolled and formed tungsten disulfide nanotubes 0.2 to 0.5 μm long and with diameters in the range from 5 nm to 37.5 nm.

Artificial self-assembled organic nanotubes were described by Ghadiri et al. They described the design, production and characterization of self-assembled nanotubes based on cyclic polypeptides [163]. They protonated these compounds, after which they crystallized into tubular nanostructures. The structures were very uniform, their length being several hundreds of nanometers, and the diameter 0.7 nm to 0.8 nm. The same team later presented an approach to the design of artificial membrane ion channels using the obtained self-assembled polypeptide nanotubes [164].

#### 4.2.6. Carbon Nanotubes

Carbon nanotubes (CNT) [165,166] belong to the wider class of artificial nanotubes (Section 4.2.5 of this text), but merit a class of their own because of their peculiarities, their importance and their widespread use in nanomembrane functionalization, as well in many other fields.

The discovery of CNT is often credited to Sumio Iijima in 1991 [167], despite the fact that almost 40 years earlier, in 1952, carbon nanotubes were observed and reported by Soviet researchers L.V. Radushkevich and V.M. Lukyanovich in the Soviet Journal of Physical Chemistry [168].

**Figure 5:**
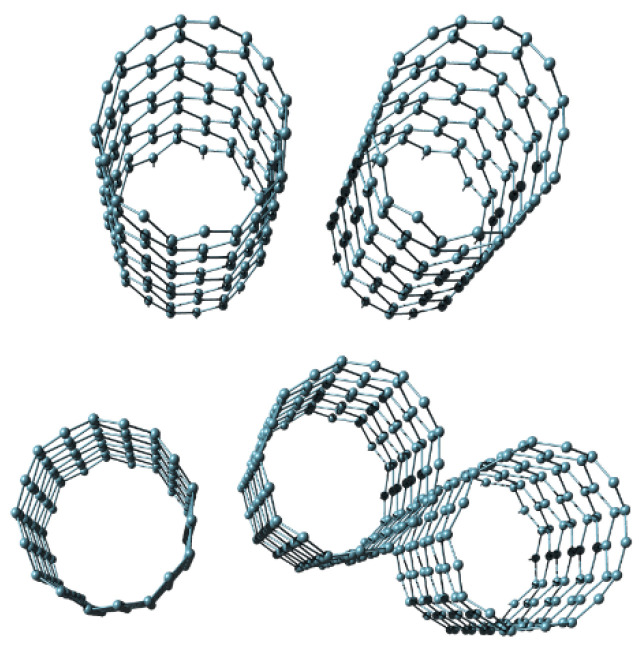
Schematic presentation of single-walled carbon nanotubes.

CNT are an allotrope of carbon and represent a hollow tube consisting of carbon atoms forming a hexagonal 2D crystal lattice (Figure 5). They may be single-walled or multiple-walled, their walls being monatomic. The thinnest freestanding single-walled CNT had a diameter of 0.43 nm, and a typical value is 1–2 nm. They have extreme aspect ratio, and actually CNTs with lengths in excess of 50 cm have been observed. Theoretically, the CNT length could be infinite. Typically, the single-walled CNTs are 5–30 nm long. CNTs may behave as metals with extremely high electrical and thermal conductivity or as semiconductors. CNTs exhibit an exceptional tensile strength.

Important from the point of view of biomimetic nanomembrane functionalization is that the inner side of CNTs is hydrophilic. This makes them useful for incorporation into nanomembranes as artificial transmembrane channels. For that purpose, they have to be relatively short and vertically aligned relative to the nanomembrane. They are used as porins in membranes for water filtration and removal of undesired ions, for instance in desalination [154].

#### 4.2.7. Antifouling Structures

The membranes of all biological cells are exposed to various kinds of external fouling based on different mechanisms. Fouling agents belong to one of the following three classes [169]: (1) passive, non-self-migratory particles and substances (for instance various external proteins, small particles of organic or inorganic matter, polysaccharides, etc.; also prions and viruses, which do not proliferate while outside the cell walls and become active and multiply only upon entering the cell); (2) passive spreadable substances (external liquids, for instance droplets of oil); and (3) active and proliferating foulants (bacteria, protozoans).

Antifouling structures and layers are essential parts of all living cells. The biological antifouling mechanisms can be roughly divided into the following groups: (1) physical structures or chemicals repelling fouling agents; (2) self-cleaning physical structures or chemicals shedding (passively removing) already present fouling agents; and (3) aggressive chemicals that actively destroy fouling agents [169,170]. Biomimetic antifouling structures can and should follow these strategies, since biomimetic membranes, especially those intended for separation applications, will be exposed to similar kinds of fouling.

##### Fouling Agent Repellents

These may include layers of superhydrophilic chemicals (e.g., hydrophilic polymers) on the membrane surface that bind a thin sheet of water (hydration layer) and thus behave as a kind of fish scales not leaving the fouling agents a place where to adhere. Another approach is the grafting of polymer brushes (nanobrushes) on the membrane surface which represents a physical method to repel a would-be fouling agent. Finally, the surface topology may be modified by making structures reject fouling agents and prevent their adhesion in the same manner a lotus leaf repels water.

##### Self-Cleaning

Self-cleaning layers and structures are intended to remove fouling agents that already found a way to adhere to the membrane. One approach is to use a layer of chemical with low surface energy (an example being perfluoro/silicon-containing polymers/macromolecules).

Active chemicals that attack fouling agents. These include metal coatings, graphene coatings, sulfides or oxides of transition metals, titanium dioxide photocatalytic nanoparticle layers and other catalytic/enzymatic agents that actively take part in destroying the foulants.

A review dedicated to imparting antifouling properties to artificial membrane surfaces was published in [169].

#### 4.2.8. DNA Transmembrane Channels

Channel structures in lipid bilayers may be formed of DNA double helix strands. It may consist of one or more DNA strands. If there are more, their incorporation into the nanomembrane is done by using the DNA origami technique [171,172]. This customizable method allows one to organize several DNA molecules in a funnel-shaped structure with a polygonal cross-section. This structure is built into the membrane orthogonally and stands upright within the lipid bilayer. The cavity within the thus-woven DNA channel represents an artificial transmembrane pore. To incorporate a DNA channel into a lipid bilayer nanomembrane, one has first to functionalize DNA. This is done by chemical modification, and in this way hydrophobic “anchors” (porphyrin-tags or cholesterol-tags) are added to DNA. After that, the DNA funnel spontaneously aligns itself orthogonally into the lipid bilayer.

The largest transmembrane channels to date were built using a DNA origami structures and their area was ten times larger than any previously man-made synthetic ion channel, comparable in size to nuclear pore complexes [173]. Its electrical conductance was an order of magnitude higher than any reported previously. The smallest DNA-based artificial transmembrane channels, consisting of only one DNA double helix, were reported in [174].

#### 4.2.9. Summary of the Artificial Structures for Nanomembrane Multifunctionalization

**Figure 6:**
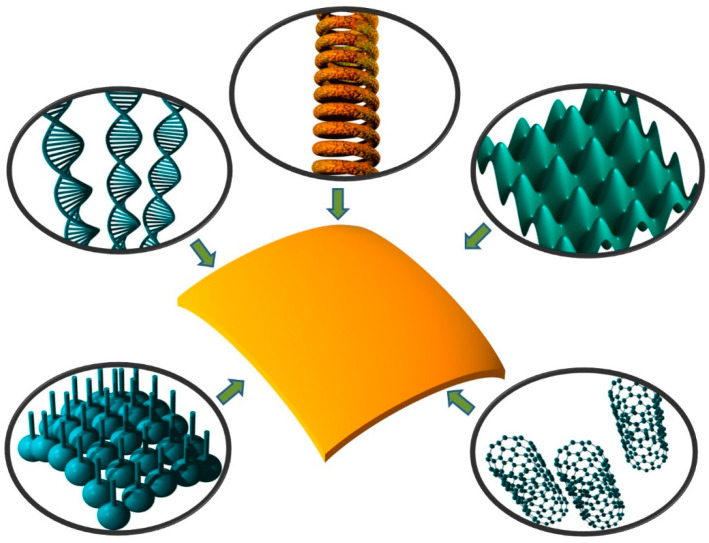
Stylized depiction of application of some artificial structures for multifunctionalization of biomimetic nanomembranes. Top left: DNA functionalization; top middle: synthetic peptide ion channel; top right: superhydrophobic surface sculpting as an antifouling mechanism; bottom left: grafted nanowires for antifouling; bottom right: carbon nanotubes for water channels.

A summarizing illustration is shown in Figure 6. It shows a choice of artificial structures for nanomembrane multifunctionalization, including spiral synthetic nanochannels, functionalization by DNA and CNT and two antifouling methods, 3D sculpting of membrane surface and grafted nanowires.

## 5. Applications

This section gives an overview of some proposed applications of biomimetic nanomembranes. The text only highlights the main fields of use, as a definitive list would be extremely long. For one, novel types of nanomembranes with many (multi)functionalities are continually being invented and developed, so it is likely that they will bring about new applications as well. As an example, if somebody makes a bionic replica of a nuclear pore complex with more functionalities than today, it could find its way into labs on a chip, and the implications for the whole genomics and proteomics would be enormous. Additionally, a bionic nanomembrane is a fundamental building block, in fact so fundamental that nature built it into basically all organisms. Thus, whole fields of science and industry could be reinvented looking through such a prism. An example would be micro- and nanosystems, where it appears as a new building block, in addition to only a few existing ones (micro/nanocantilever, microbridge, diaphragm, etc.). So, the list is necessarily open, and new applications will be appearing as you read this text. A summative table reviewing some applications of biomimetic nanomembranes classified by their fields of use can be found below (Table 3).

### 5.1. Active Nanofluidic Devices Based in Ion Transport

Nanofluidics [49] investigates the transport of fluids and ions at the nanometer scale [175]. In this way it is directly connected with basically all nanopore-based mechanisms of biomimetic multifunctionalization. In this moment, one can say that the development of nanofluidics is in full swing.

Various active devices have been developed, akin to their micro/nanoelectronic counterparts, but based on the transport of ions instead of electrons and holes. These start with ion pumps, which incorporate some nanofluidic-based diodic behavior [151], continue with nanofluidic diodes [176,177], bipolar ionic transistors [176], and nanofluidic field effect transistors (FET), which were theoretically conceptualized in [178] and practically implemented in [179]. More recent implementations include [180,181]. The operation of active nanofluidic devices is based on the fact that in nanofluidic channels, the surface charges usually have larger effects on the flow of ions than to the flow of liquids, in contrast to the phenomena observed in microfluidics. This is a consequence of the Debye length, the distance scale for ion screening. Thus, the nanofluidic ionic current can be tuned by modifying the surface charge density when the electrical double layers in the channel do not overlap [176].

One might argue that the speed of contemporary active nanofluidic devices is much lower than that of their electronic counterparts. On the other hand, a counterclaim may be found in the fact that all our neural processes are based on nanofluidic ion transport. Hence, we are talking about a process that brought to this development the most sophisticated micro- and nanostructured biological machines that ever existed, namely our brains. This proves the point that there is much room for further biomimetic development. In 2020, Bocquet proposed [175] the development of ionic machines inspired by the functioning of our neuronal systems, including building synthetic neuromorphic biomimetic computer elements.

### 5.2. Two-Dimensional Nanofluidics

Koltonow and Huang proposed the idea of fluid flow confined to 2D space [182] as a parallel to 2D charge carrier transport in quasi-2D sheets (graphene, MXenes, generally nanomembranes). For this purpose, they proposed making sandwiches of nanomembranes/monolayers (e.g., graphene) at a distance smaller than the Debye lengths of the surrounding solid nanosheets. The interspace between the sheets is filled with fluid, and there are pores in the sheets that enable transport between different “levels”. In a situation similar to semiconducting 2D systems, the confinement of fluid into 2D nanochannels should ensure novel physical effects in such 2D fluid sheets, mainly vastly increased conductivity of cations.

### 5.3. Biosensors

The field of nanomembrane-based biosensors is very wide, and it opens pathways to extensive and numerous practical uses. Many different types have been proposed to date, of which we give an overview of a few.

A combination of biomimetic nanomembranes with plasmonics and metamaterials has found use in ultrasensitive chemical or biological sensors [27,183,184]. These sensors are refractometric and affinity-based (using the effect of selective adsorption and desorption). They typically consist of metal-dielectric nanocomposites, ordered in a form of 2D arrays. The presence of adsorbed analyte at the metal-environment interface modifies the effective refractive index near the surface of the nanoplasmonic structure or metamaterial (which may be a freestanding metasurface [60] or a bulk structure, the so-called Catalyst Plus sensor [185]). In this way, the propagation of surface electromagnetic waves (evanescent waves) is tuned proportionally to the amount and species of the analyte. Such sensors exhibit extreme sensitivities, even reaching a single-molecule accuracy [17], dependent on the nanostructure used. Biosensors that use ion channel switches were described by Cornell et al. [24].

More recently, Medina-Sánchez et al. described a high-performance DNA detection biosensor that recognized DNA strands of avian influenza virus H1N1 [186]. Their sensor was based on rolled 3D nanomembranes. The authors proposed the use of their sensor for detection of proteins and enzymes, as well as for monitoring of in vivo cell behavior.

El Afandy et al. described a nanomembrane-based sensor of thermal diffusivity and conductivity [18]. Their technique is based on the increase of phonon-boundary-scattering rate of nanomembranes. They showed it on a single cell. The low flexural rigidity and softness of nanomembranes ensure a close contact with the cells, independently on the irregularity of their shapes. The method enabled a novel biomedical diagnostic technique, since it can detect differences between, e.g., different types of cancer cells.

### 5.4. Renewable Energy

#### 5.4.1. Fuel Cells Based on Proton Exchange Membranes

Conventional proton exchange membranes (PEM)-based fuel cells [23] (also denoted as polymer electrolyte membrane fuel cells) customarily use 50–150 μm-thick Nafion^®^ (a copolymer of tetrafluoroethylene with sulfonic acid-terminated perfluoro vinyl ether) membranes to ensure proton conduction. Membranes made of other materials are also used sometimes, for instance Gore-Select™ mechanically reinforced membranes which are much thinner (about 10 μm). PEM fuel cells operate at low temperatures (below 100 °C), are easy to scale up, offer high power densities and high efficiencies, while at the same time emitting zero amount of greenhouse gases (their only exhaust is pure water). Today they are already being used as safe power sources for hydrogen-fueled cars (Toyota Mirai, Hyundai Tucson, etc.). Other practical uses include portable devices like laptops, smartphones, tablets, etc. Extensive research efforts are currently directed toward the development of improved components for PEM fuel cells, with a large portion of these efforts being spent on researching novel types of membranes.

Their operation is based on two mechanisms: vehicular transport where hydrogen ions (H^+^) diffuse in the form of hydronium ions (H_3_O^+^); and the Grotthuss mechanism (“proton jumping”) in water wires, where H^+^ ions hop between neighboring water molecules in a string within nanochannels spanning the membrane. For a proper operation, a PEM fuel cells must be kept hydrated at a percentage within required limits.

In this text, we briefly describe approaches to replace the conventional Nafion^®^ membranes with much thinner biomimetic nanomembranes functionalized by nanopores acting as water channels. Their advantage, apart from vastly increased packaging density and thus available power per unit volume, would be a much faster proton transport; instead of labyrinthine ways for water channels as in the conventional membranes, they require only short and straight biomimetic nanopores, which should furnish proton transport rates comparable to those in biological cell walls and much higher than in Nafion^®^. The mechanism is here also a combination of the vehicular one and Grotthuss effect [187,188]. As far as the artificial pores are concerned, a convenient choice would be short and vertically aligned carbon nanotubes whose interior is hydrophilic, so that water wires are spontaneously formed within them, ensuring proton transport. Many polymer hosts are available for nanomembranes for PEM, including but not limited to amorphous fluoropolymers and polybenzimidazole. The membranes would need to be reinforced, e.g., by lamination with an inorganic layer.

#### 5.4.2. Solar Cells

Biomimetic nanomembranes have been used in stretchable and curved electronics. Silicon single-crystalline nanomembranes have been proposed for the use in solar cells [57], due to the excellent performance of silicon single crystal compared to amorphous silicon or polysilicon. Single crystals are very expensive in comparison, but this sole advantage of the other types of silicon solar cells becomes unimportant when minute amounts of material are used, as is the case with nanomembranes. The use of silicon microcells on foldable substrates has been proposed. The performance of these solar cells can be further enhanced by e.g., lenticular optical concentrators, antireflection structures and other optical enhancement techniques [189].

Sculpted nanomembranes in a rolled-up geometry have been proposed for use in both infrared detectors and solar cells [190], due to their improved light trapping properties enabled by the rolls.

#### 5.4.3. Hydrogen Economy—Water Splitting

Water splitting to hydrogen and oxygen represents a crucial step towards a hydrogen-based clean economy. Nanomembranes can be used to make ultra-compact catalytic microreactor cells to perform water splitting using solar energy (photocatalysis) [191]. For this purpose, titanium dioxide, the well-known material for catalytic water splitting, was used. Another approach to solar-induced photocatalytic water splitting is to use composite organic-inorganic nanomembranes laminated with photocatalyst or, alternatively, filled with its nanoparticles.

Silicon diphosphide (SiP_2_) and silicon diarsenide (SiAs_2_) nanomembranes have also been proposed for highly efficient photocatalytic water splitting [192]. Both materials promise high carrier mobilities and good mechanical, thermal and dynamic stabilities. Another pair of alternative materials for the same purpose are beryllium nitride (BeN_2_) and magnesium nitride (MgN_2_). Monolayers of these materials as water-splitting photocatalysts were considered in [193]. Janus monolayers (i.e., with different front and back side, “two-faced”) of metal chalcogenides Ga_2_XY and In_2_XY (X = S, Se, Te; Y = S, Se, Te) have also been described as promising materials for the same purpose [194].

Instead of using a photocatalytic structure, an alternative approach is to use electrocatalyst nanomembranes [195]. Wang et al. used nanomembranes with reduced graphene oxide combined with NiO/Ni. Alternatively, metallic Ni_3_S_2_ ultrathin sheets fabricated by atomic layer deposition proved themselves efficient and stable electrocatalysts for this purpose [196].

### 5.5. Nanomembrane Separation

Porous nanomembranes are often used as separators or molecular sieves, filtering various kinds of fluids (both liquid and gaseous) in a great many applications. Their pores may belong to some of the previously described classes (ion channels, ion pumps, nuclear pore complexes), but do not have to. Simple nanometric openings are convenient for many of applications in separation. The necessary requirement, however, is that the size of pores across the membrane is uniform. The nanomembranes do not even have to be porous—structures based on diffusion through non-porous synthetic nanomembranes are also used.

First, we classify some mechanisms used in filtration that are applicable to biomimetic nanomembranes. A possible classification is presented in Table 4.

It may be useful to quote here the definitions of pores according to IUPAC: micropores have diameters below 2 nm. Mesoporous materials have pores 2–50 nm, while macroporous materials have pores with diameters above 50 nm [197]. Unofficially, all pores with nanometric dimensions are denoted as nanopores; their diameters may range from the order of 0.3 nm to about 20 nm.

Membrane separation science is an enormous field, a science in itself, with numerous related technologies, processes and methods [198,199]. Basically, in a lot of fields where conventional membranes are used, their place could be filled by biomimetic nanomembranes. The questions of mechanical strength can be solved, instead of using porous substrates to mechanically support the active part of the separation membrane, by applying porous nanomembranes functionalized with nanofillers (e.g., woven CNT, zirconia fillers, etc.) that may drastically increase their strength and avoid the need for porous substrates altogether. What we are left with are structures that basically perform the same function, but with at least several orders of magnitude decreased space requirements. Additionally, the throughput of nanomembranes would be vastly higher than that of their micro or macro counterparts, because instead of having to pass through drastically longer and meandering separation channels, the filtrates would have to pass only a nanometer-long and straight pathway that actively rejects the undesired agents and species. Additionally, nanomembranes can be functionalized with target-oriented biomimetic pathways like ion channels, ion pumps and even simplified nuclear pore complexes. In Table 5 we present some applications of biomimetic nanomembrane separation.

Without any claims for exhaustiveness, we describe in the next subsections a choice of some areas where the use of biomimetic nanomembranes has been notable. This is only intended as an illustration of the much wider field of applications of biomimetic nanomembrane in separation science and technology.

#### 5.5.1. Remediation and Environmental Protection

Environmental uses of nanomembranes include waste water treatment, removal of inorganic and organic pollutants, especially the persistent ones (e.g., dioxin, radioactive nuclides, pesticides, etc.). Nanotechnological methods for water remediation include three approaches: the use of nanomembranes, nano-adsorbents and nanocatalysts. The application of functionalized nanomembranes for environmental protection and remediation was reported in [200]. It appears that the optimum results are obtained if nanomembranes are multifunctionalized by photocatalytic nanoparticles and thus perform a dual role of simultaneously filtering some pollutants while actively destroying others. An essential functionality of membranes for the use in remediation and wastewater treatment is imparting the antifouling properties at the “dirty” side.

#### 5.5.2. Food and Beverages

Nanomembranes in the food industry find applications in removal or photocatalytic/enzymatic destruction of viruses, bacteria, aflatoxin, cyanotoxins and other toxins, pesticides, bisphenol and other undesired contaminants, also for filtering inorganic substances including heavy metals, etc. Among examples are bioconversion and removal by separation of lactose in milk and dairy products, obtaining cheese and other fermented products from milk, filtering beer from mother solution, etc. [201]. Other uses of biomimetic nanomembranes in the food and beverage industry include nanomembrane-based sensors for in situ monitoring of various critical ingredients that contribute to the overall quality, also the detection of the above mentioned dangerous, cancerogenic and even potentially lethal contaminants.

#### 5.5.3. Desalination and Potable Water Production

One of the often-encountered approaches to water treatment to remove sodium chloride and other minerals from seawater and to make it potable are nanoporous nanomembranes functionalized by aquaporins channels [9,202]. Usually structures with a porous support are used to ensure mechanical integrity. Nowadays, the use of aquaporins for desalination is becoming a profitable industry. Another approach is to use carbon nanotube-based water channels for the same purpose [203,204]. It has been shown that water moves through carbon nanotubes exceptionally fast (“hyperlubricity”). Other approaches includee nanoporous graphene layers [205].

### 5.6. Biomedical Applications

#### 5.6.1. Two-Dimensional Scaffolds for Tissue Regrowth

The main goal of tissue engineering is to repair, regenerate and restore tissue damaged or degenerated due to, e.g., disease or injury including mechanical or toxic trauma to its original form as fast and safely as possible. A very important role in this task belongs to artificial scaffolds whose role is to stimulate and sustain the regrowth and proliferation of new cells, which is a very complex process by itself. To facilitate regrowth of cells, cell systems, tissues and even whole organs, one typically uses a 3D or 2D structure (the scaffold) to serve as a template for cell growth, to which one seeds the desired cells. The follow-up may be that one adds the necessary growth factors, applies some kind of external biophysical stimulus, or performs both of these procedures [16]. The whole regrowth process may occur in vitro, in vivo, or combined.

While designing a scaffold to facilitate the initial steps of regeneration and repopulation of the differentiated cells, including mandatory revascularization, one of course must take care of biocompatibility (a scaffold must not cause an inflammatory process or tissue rejection and must not release any toxic components either directly or indirectly (through the process of its intermediate or final decomposition or resorption by the organism). Ideally, it will remain in the organism as long as necessary and then it will spontaneously break apart and be dissolved (biodegradability).

Various tissues can be engineered in a biomimetic manner, from bones [206,207] and cartilage [208], over muscle cells and skin (including hair follicles) [209], cardiovascular tissues including heart muscle tissue [210,211], internal organs (e.g., liver, pancreas, intestines, kidneys), [212] airways in lungs, to highly complex neural tissues [14,16,213]. The implemented scaffolds must be mechanically compatible with the surrounding tissue and at the same time ensure easy handling. Their structure must be porous and ensure easy adherence of the cells to be regrown. The pores should be interconnected to allow a further cell growth and spreading in a manner that maximally corresponds to the natural one.

Two main kinds of materials are usually used for tissue engineering scaffolds, ceramics and polymers (either natural or artificial ones) [16]. In addition to these, one sometimes uses a combination or composite of both. Ceramics are often used for bone regeneration and include materials like hydroxyapatite or tri-calcium phosphate. The most popular natural polymers are chitosan and collagen, while there is a host of synthetic polymeric scaffolds like, e.g., hydrogels, polystyrene, copolymers, acrylates, polyglycolic acid, etc.

Among various scaffolds for tissue regrowth used to date, there is a group of 2D scaffolds including synthetic nanomembranes that ensure 3D cell growth. The materials for these nanomembranes include the above-mentioned ones [214], but also novel structures like carbon nanomembranes and graphene, polycaprolactone and many more [215,216,217].

**Figure 7:**
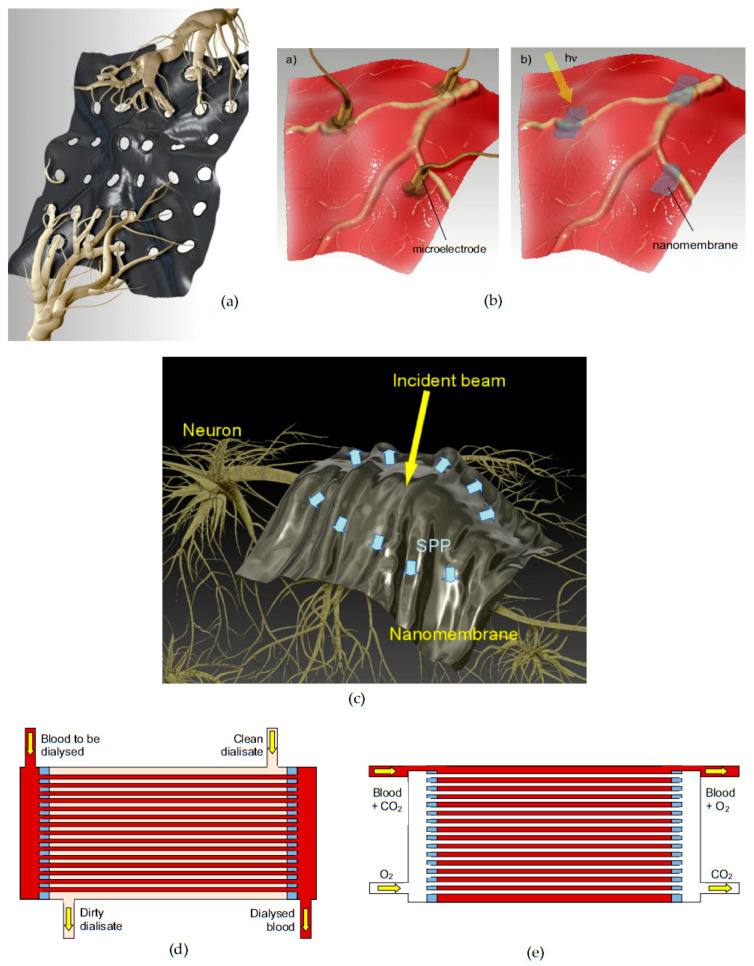
Summary of biomedical applications of biomimetic nanomembranes. (**a**) Simplified depiction of metal-composite nanomembrane sieve bypassing a nerve damage, with partially re-grown neural tissue. Authors’ own work from [52]. (**b**) Comparison of conventional and nanomembrane-based in vivo neural interfacing. Left: Conventional implanted electrodes. Right: non-invasive injected soft plasmonic nanomembrane-based electrodes, interrogated by modulated infrared beam. Authors’ own work from [52]. (**c**) Structure of a nanomembrane-based neural interface with coupling based on long range surface plasmons polaritons. Authors’ own work from [52]. (**d**) Schematic presentation of the operation of the artificial kidney. (**e**)

Wu et al. [215] investigated the biocompatibility and biotoxicity of graphene as well as its potential uses in tissue regeneration and regrowth, as well as its role in cell reorganization and regrowth. Jakšić et al. considered a possible use of metal-composite nanomembranes as 2D scaffolds for nerve regrowth [52]. An analysis of the use of biomimetic nanomembranes as a ultrathin extracellular scaffold for free-standing cell sheets is given in [15]. Figure 7a shows a simplifiedpresentation of a metal-composite nanomembrane sieve as a nerve cuff bypassing a nerve damage.

#### 5.6.2. Neural Interfaces/Neural Cuffs

We considered the feasibility of using functionalized nanomembranes as a minimally invasive link connecting external optical signals and neural tissue, using long-range surface plasmon-polaritons (SPP) [218,219]. Neural activity was already recorded in vitro, using coupling of propagating free-space modes with neural impulses by way of long-range SPP, with excellent sensitivity.

We continue by describing a potential use of a metal composite nanomembrane as an implantable “nerve cuff” electrode that may be positioned around the epineurium as a kind of “shrink-wrap”, with tens of nanometers thickness, which ensures intimate contact with the neural tissue [52,220].

When conventional microelectrodes are inserted (Figure 7b, left), they damage capillaries, extracellular matrix and glial and neural cells. The results are inflammations, swelling, bleeding, necrosis, etc. In the long run, glial cells try to phagocytose the foreign body, damaging it and isolating it by a few hundreds of µm of scar tissue.

The bulk metal of a conventional microelectrode is replaced here by a metal nanomembrane with a thickness three orders of magnitude smaller than a bacteria size, making the latter outstandingly soft and flexible at macroscopic level and thus minimally invasive; such “soft” biomimetic electrodes will cause minimal mechanical damages (Figure 7b right). An illustration of surface-plasmon polariton soft nerve cuff for the readout of neural activity is shown in Figure 7c.

A previously described method of nanomembrane insertion [51] (Section 2.2 of this text) is the injection by a simple syringe, which makes use of their surprising robustness and toughness, as well as of their extreme foldability.

Excitation and readout could be done by modulated infrared laser beam, since it propagates well through living tissue. The surface relief of the semi-crumpled membrane ensures coupling of propagating with evanescent modes. Additionally, it has previously been shown in this text (4.1.1) that nanomembranes can be made fully biocompatible through proper material choice and functionalization through both-sided lamination.

#### 5.6.3. Wearable Artificial Kidneys

The development of wearable artificial kidneys [221,222] is of great interest for patients with kidney failure (chronic kidney disease and end-stage renal disease). The need to keep the patients connected to large systems for dialysis for prolonged periods of time is disabling, drastically altering their life, the procedure is slow and painful, and abrupt changes in blood composition may cause a shock to the patient (the so-called dialysis washout), requiring at least hours to recover. On the other hand, prolonged periods between dialysis sessions may leave the patient with increased levels of toxins in blood. A wearable device for dialysis would ideally allow for continuous detoxification and at the same time permit the patient to be mobile while under treatment, thus drastically improving his/her quality of life. Most contemporary portable devices weigh somewhere between 3 and 30 kg [223]. Because of this, one of the key targets of contemporary microfluidics and microelectromechanical systems (MEMS) technology is the development of wearable or even implantable kidneys through a decrease of their dimensions and an increase in efficiency [224].

A simplified principle of operation of an artificial kidney (renal replacement device) is shown in Figure 7d. Its main component is a system of polymer or copolymer hollow microfibers with nano-sized pores. The patient’s blood flows through the microfibers, which are surrounded by dialysate, is filtered through the system of pores, and waste components such as urea and creatinine are removed and washed into dialysate.

There are several experimental systems that mimic the toxin removal function of natural kidneys and have been scaled down for possible use in wearable systems. Such systems are based on the use of inorganic porous nanomembranes and have been reported by Hill et al. [225]. They used silicon nitride membranes with a thickness below 100 nm and with nanometer-sized pores. Such nanomembranes showed high filtration performance while at the same time exhibiting excellent robustness. They reduced the necessary membrane area for hemodialysis compared to conventional, floor-standing equipment by 100 times or even more. This may enable the production of miniaturized systems that could lead to hemodialysis at home, with longer duration and with improved removal of toxic components from blood. Thus, nanomembranes represent an important enabling step towards wearable artificial kidneys, potentially ensuring continuous blood purifying through ultrafiltration hemodialysis [226].

#### 5.6.4. Portable Artificial Lungs

State-of-the-art artificial lungs aim to enhance the level of oxygen exchange in the blood of patients with lung failure [227,228]. To this end, the devices use large bundles of hollow fibers made of rolled porous membranes with typical radii of 100 to 200 μm. Oxygen fills the space inside of each microfiber, while blood flows around the fibers. Diffusion ensures flow of oxygen through pores into the blood, while simultaneously the same mechanism ensures that carbon dioxide exiting the blood enters the microfluidic fiber. A simplified principle of operation of the artificial lungs is shown in Figure 7e.

The critical goals in the fabrication of the artificial lungs are to minimize the dimensions of the hollow fiber bundle for gas exchange (since it represents the most part of the bulk of the artificial lungs), and at the same time to maximize the gas throughput. Both of these ensure a decrease in the dimensions of the structure and thus contribute to improved mobility of the patient while on artificial lungs. Typically, biocompatible polymer materials are currently used for the hollow fibers, for instance polypropylene [227].

Bearing in mind the dimensions of nanomembranes, they seem ideal for both of the main improvement goals—since gas has to cross a much shorter path through the pores than in the conventional thick membranes, throughput will be enhanced. Simultaneously, the fiber walls will be much thinner, thus contributing to the improved portability of the whole system.

#### 5.6.5. Selective Drug Delivery and Therapy

The pharmacokinetic behaviors of drugs is closely related to cell membranes. A majority of therapeutic drugs target either receptors within membranes or organelles and their parts like chromosomes/DNA within cells, i.e., they interact with biological membranes either by acting upon their built-in blocks or passing through them using some of their existing pathways for matter exchange (for instance, membrane proteins that assume the role of transport pumps, or ion channels) to proceed to targets inside the cell or even inside organelles.

Nanoparticles can be used for medical treatment in several manners [229]. The oldest approach among them is their use as vehicles to carry drugs and therapeutic agents (diagnostic tools for marking the affected tissue, mediator agents for photodynamic therapy, chemotherapy, gene therapy, etc.) to their target [230], which is often cancerous tissue [231]. A variant of this approach is to “hide” therapeutic agents in freely movable cells, thus using them as carriers [232]. Another method is direct introduction of, e.g., metallic or titanium dioxide nanoparticles into the affected tissue as mediator agents for hyperthermia treatment. For this purpose, one causes heating of nanoparticles by remote tissue-penetrating electromagnetic radiation, e.g., laser [233] or microwaves [234], and the hot nanoparticles cause thermal death of the heat-sensitive cancer cells [235]. Plasmonic effects are used for this purpose too [232]. Finally, one of the approaches to drugs delivery (e.g., antidiabetic, antihypercholesterolemic, etc., drugs) is the direct injection of their nanoparticles, without a carrier [236].

Problems with all of the quoted approaches are the poor targeting of the affected tissue due to bodily fluid circulation in vivo, a short life of nanodrugs in the living tissue, and poor penetration depths. This is the reason why only a few carrier-based nanoparticle drugs have been approved by Food and Drugs Administration [232].

**Figure 8:**
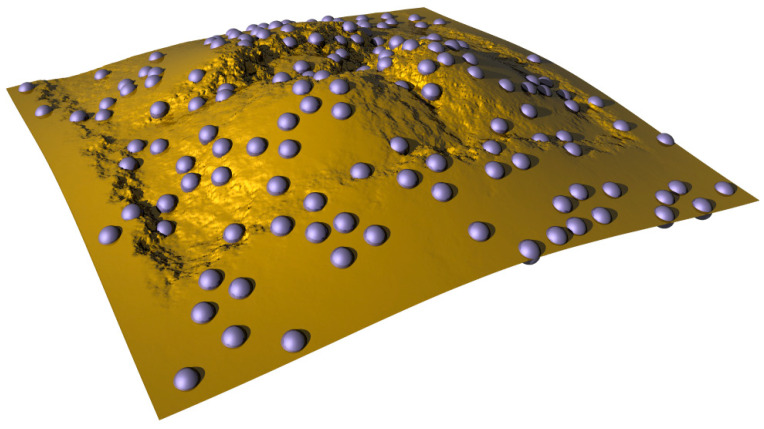
Drug nanoparticles (violet spheres) embedded in nanomembrane (golden) as a drug delivery system.

Biomimetic nanomembranes could be used as carriers for nanodrugs and other therapeutic agents [19]. Whether the drugs are in the form of nanoparticles or not, their uncontrolled release and spreading through the body could potentially be harmful and toxic. However, if they are embedded in a biomimetic nanomembrane functionalized to ensure biocompatibility, then the whole ensemble functions as a soft macroscopic carrier with nano-volume and with very good targeting. It is necessary to ensure the adherence of the unfurled biomimetic nanomembrane to the treated tissue in the position where the nanomembrane was injected. In this case, the drugs will be concentrated in exactly the position where the soft nanomembrane is located. In an ideal case no free nanoparticles will roam the organism. Furthermore, the active area of nanomembranes is close to the theoretical maximum (“the interface without volume”); thus, they offer high exposure of the tissue to embedded nanoparticles and maximized efficiency combined with localization (Figure 8). The critical steps here represent possible decomposition and resorption of the nanomembrane under the influence of body fluids and biological processes and subsequent potential cytotoxicity [237].

## 6. Future Outlook

Here we briefly present a few of possible directions for future research. There is a vast number of such directions that we do not mention here, and the area keeps expanding on a daily basis. The topic of biomimetic nanomembranes opens literally endless horizons for both research and applications.

### 6.1. Novel Types and Architectures of Artificial Pore Complexes

One field that leaves much to be desired is the fabrication of artificial ion channels, ion pumps and nuclear pore complexes. Structurally, all types of ion-transporting pores are currently far less complex than their living counterparts. Functionally, many artificial ion channels are close to the biological structures. Ion pumps require much more research, and it will require a lot of effort to achieve both the functionality and complexity of the natural structures. A similar claim, only on an even larger scale, can be made for pores located in the membrane around the cell nucleus. Huge advances have been made in recent years. Yet larger ones remain to be achieved.

### 6.2. “Living” Plasmonics and Metamaterials

One promising direction of research is the integration of plasmonics with functionalized biomimetic membranes. The use of nanomembranes for plasmonics and metamaterials was considered in [27]. A further logical direction would be research into biomimetic plasmonics. Publications in this promising area are almost nonexistent. One of the rare papers in the field investigates sensors of tumor cells using surface plasmon resonance functionalized by artificial lipid bilayer membranes [238]. Even more scarce are publications on biomimetic metamaterials.

In this subsection we consider the possibility of researching nanomembranes with plasmonic and/or metasurface properties functionalized by some of the biomimetic structures previously considered in this text. Functionalization of nanomembranes made of metal or some other plasmonic material using synthetic ion channels, ion pumps, water channels or artificial nuclear pore complexes seem to be especially promising for the field of biological sensing. Additionally, all plasmonic nanomembrane-based sensors could benefit from antifouling functionalization which could improve their applicability in the real field conditions.

### 6.3. Quantum Functionalities

Nanomembranes represent quasi-2D objects, which means that charge carrier transport in them will obey the rules of low-dimensional systems and thus will show marked quantum mechanical effects. This in itself is a large field of investigation, since each low-dimensional material will have its own set of properties and peculiarities, including effects rarely or never observed in nature. It may be said that the topic of combining biomimetic nanoobjects and quantum physics is in its very infancy, although the results to date are encouraging and have already brought us to a host of novel effects and even novel and improved electronic devices [48].

### 6.4. Meta-Bilayers Adding Functionalities beyond Natural to Biomimetic Nanomembranes

The possibility of using an extended toolbox of materials compared to biological structures opens the path to imparting new functionalities. For instance, one can make lipid bilayers in which proteins are not the main functionalizing agents. Various alternative functions could be imparted to them, including incorporation of particles with magnetic, optical, plasmonic, catalytic including photocatalytic, pharmaceutical and other properties. This would make “meta” lipid bilayers out of them—those with properties that go beyond the natural ones.

### 6.5. Shape-Shifting Nanomembrane Bulges with Active External Control

The curvature and the overall profile of spatially sculpted nanomembranes could be externally controlled. It is known that several macroscopic properties of micro and nanosurfaces are influenced or even defined by their surface shapes—examples include self-cleaning and superhydrophobic behavior, actually a drop-repellent property (the lotus leaf effect) [239,240], and drop-pinning on hydrophobic surfaces (the rose petal effect) [241]. An active control of the surface microstructure could switch between these two modes of superhydrophobic behavior. Another property dictated by the shape of the surface details is adhesion, observed in some organisms ranging from simple bacteria to such sophisticated organisms as some frog species (e.g., Litoria caerulea), where the active control over the adhesive properties is obtained through the change of the surface shapes. A practical approach to control over the adhesion properties of membranes was considered in [242] for the case of thick elastic membranes. The actuation needed for the surface relief change was obtained through pneumatic mechanism.

In this work we extrapolate the active approach to shape shifting to the field of nanomembranes, which, being much thinner than the conventional membranes, could ensure a more energy-efficient and precise switching between different modes of hydrophobicity and hydrophilicity, as well as dynamical control over adhesive properties. The actuation mechanisms that could be used for such shape shifting include applied electric field, single-sided pressure, mechanical change of the membrane support through stress/stretching or bending, the external optical field, etc.

## 7. Conclusions

In this paper, we considered the main properties of biomimetic synthetic nanomembranes, and their fabrication and functionalization. We also considered the main biological functional parts of cell nanomembranes, the ones ensuring basic complex functionalities that make up life processes, including membrane ion channels and ion pumps, as well as parts of the cell nucleus envelope, the nuclear pore complexes. After that we proceeded to consider their biomimetic counterparts. One of the important functionalities that we considered is imparting the biomimetic antifouling properties to the membranes. In the next part we gave an overview of selected applications of biomimetic nanomembranes, either theoretically proposed or actively used. At the end we gave some extrapolations and possible directions for future research.

It was our aim in this review to stress the importance of functionalized synthetic biomimetic nanomembranes within the context of modern nanoscience and nanotechnologies. We hoped to highlight the importance of the topic, as well as its profound applicability potential in many facets of human life.

Biological nanomembranes are the most ubiquitous nanomachine in nature, which says a lot about their usefulness and versatility. Mimicking their functions and complexities offers a cornucopia of rewards. It is only up to us how deep we will be able to reach.

## Figures and Tables

**Table 1:** Classes and types of synthetic nanomembranes based on membrane material.

Classes of Synthetic Nanomembranes	Types Belonging to the Class
Inorganic nanomembranes	Metal nanomembranes Metal composite and alloy nanomembranes Diamond nanomembranes Diamondoids Diamond-like carbon (DLC) nanomembranes Semiconductor nanomembranes—single element Semiconductor nanomembranes—compounds Freestanding monatomic sheets (incl. graphene) Freestanding inorganic monomolecular sheets
Hybrid organic /Inorganic	Interpenetrated structures Metal–organic frameworks
Organic nanomembranes	Single-polymer (pure) Copolymer (blended) Carbon nanomembranes (CNM)
Synthetic biological nanomembranes	Model lipid bilayers (black lipid membranes, painted bilayers, synthetic lipid bilayers)

**Table 2:** Generic strategies for the fabrication of biomimetic nanomembranes.

Substrate	Fabrication Strategies	Particular Approach
Solid	Sacrificial structure	Top-down (thin film technologies)
	Exfoliation	Mechanical or chemical delamination
Liquid	Gas–liquid interface	Bottom-up (self-assembly) Thin film technologies
	Liquid–liquid interface	Self-assembly

**Table 3:** Some select applications of biomimetic nanomembranes classified by fields of use.

Application Field	Application Type
Environmental Protection	(1) Air pollution control—removal of pollutant particles and volatile compounds from airstreams (2) Wastewater treatment—removal of pollutants and recycling (3) Remediation
Biosensing and chemical sensing	(1) Ultrasensitive chemical, biochemical and biological sensors (2) Simultaneous sensing of multiple analytes by a single sensor
Toxicology, forensics and homeland defense	(1) Recognition of toxic inorganic, organic and biological agents (2) Removal of toxic agents
Renewable energy & power industry	(1) Fuel cells (2) Solar cells (3) Water splitting (4) Micro-power sources and microbattery arrays (5) Nuclear fuel production, purification and enrichment (6) Hydrocarbon fractionation (7) Environment-friendly fuel purification (e.g., desulfurization)
MEMS/NEMS	(1) Active nanofluidic devices based on ion transport (2) Self-healing micro and nanostructures (3) Stretchable and foldable devices (4) Very high frequency microoscillators and microresonators (5) Catalytic membrane microreactors (6) High temperature microreactors (7) Smart Labs on a chip
Molecular sieves and Separators	(1) Water, oil, gas separation from undesired ingredients (2) Removal of heavy metals (3) Reclaiming of precious materials including noble metals (4) Desalination
Biomedical applications	(1) Two-dimensional scaffolds for tissue regrowth (2) Biointerfaces including neural interfaces (3) Wearable and implantable artificial kidneys (4) Portable artificial lungs (5) Drugs delivery and disease control (6) Pathogenic bacteria, viruses, prions recognition and deactivation
Bioengineering and genomics	(1) DNA analysis/separation/replication (2) Gene sequencing, genomics (3) Cell biotechnology (4) Biomarker detection
Food and drinks industry	(1) Food and beverages purification (2) Potable water production through purification
Chemical Engineering	(1) Multicomponent gas mixtures separation (2) Gas dehydration (3) Separation of liquid chemicals (4) Chemicals analysis (5) Microfiltration, ultrafiltration and nanofiltration (6) Environmentally friendly petrochemistry

**Table 4:** Classes and types of filtration by biomimetic nanomembranes.

Class	Type	Mechanism and Action
Pore filtration	Particle filtration	Pressure-driven filtration through >1 μm pores and above, removes large suspended particles ^1^
	Microfiltration	Pressure-driven filtration through 0.1 μm to 1 μm pores, removes bacteria and suspended organic and inorganic particles
	Ultrafiltration	Pressure-driven filtration through 1–100 nm pores, removes all above plus viruses
	Nanofiltration	Pressure-driven filtration through ~0.3–1 nm pores, removes all above plus large (polyvalent) ions
Filtration by diffusion	Reverse Osmosis	An applied pressure is used to overcome osmotic pressure for separation through a semi-permeable membrane, removes all above plus small (monovalent) ions
	Forward Osmosis	Separation driven by osmotic pressure gradient through a semi-permeable membrane
	Dialysis	Solute separation is induced by the difference in solute diffusion transport through the membrane
Filtration assisted by liquid-gas phase transition	Pervaporation	Separation of mixtures of liquids by permeation through a membrane, followed by vaporization
	Membrane Distillation	Thermally driven separation of liquids where only vapor molecules move through a microporous hydrophobic membrane
	Gas Permeation	Separation of gas mixtures permeating a membrane based on the fact that the flux of each gas is different
	Evapomeation	Separation of mixtures of liquids by full vaporization through a non-porous or porous membrane

^1^ Only applicable if the biomimetic nanomembrane is supported by a porous substrate.

**Table 5:** Some applications of biomimetic nanomembrane separation.

Class	Type	Action
Water treatment	potable water	removal of organic pollutants, arsenic, ammonium, manganese, iron, etc.
	process water	removal of all process pollutants, especially heavy metals incl. lead, mercury, also dyes, organic by-products, aromatic hydrocarbons…
	waste water	removal of contaminants, including bacteria, viruses, cyanobacteria, protozoans, organic and inorganic pollutants
	remediation	removal of contaminants, including toxins, heavy metals and radioactive nuclides
	chemical spillage	removal of contaminants
	concentration of trace ingredients	harvesting of e.g., noble metals from seawater, present in it in trace amounts
Food and beverages treatment	food ingredients	removal of biological and other pollutants, sterilization
	sugar, oil production	organic and inorganic material removal
	dairy products	biological contaminants removal
	wine production	filtering, contaminants removal
Process industry	chemical processing	undesired species removal, refinement
	petrochemistry	separation of undesired compounds from liquids, gases
	fertilizer production	removal of organic and inorganic contaminants
	pulp and paper mills	refinement
	pharmaceutical industry	removal of organic and inorganic contaminants, refinement
Power industry	refining of biofuels	refinement
	refining and fractioning of natural oil	hydrocarbons separation, desulfurization, refinement
	refining of natural gas	hydrocarbons separation, removal of N_2_ and CO_2_, refinement
	fuel cell membranes	water wires in PEM structures (incorporation as an integral part)
	batteries production	removal of organic and inorganic contaminants, refinement (incorporation as an integral part)
